# Potential impacts of environmental bacteria on the microbiota of loggerhead (*Caretta caretta*) and green (*Chelonia mydas*) sea turtle eggs and their hatching success

**DOI:** 10.1002/mbo3.1363

**Published:** 2023-06-05

**Authors:** Colleen M. McMaken, Derek A. Burkholder, Rosanna J. Milligan, Jose V. Lopez

**Affiliations:** ^1^ Halmos College of Arts and Sciences Nova Southeastern University Florida Fort Lauderdale USA

**Keywords:** beach sand, eggs, hatching success, *Pseudomonas*, sea turtle

## Abstract

Sea turtle hatching success can be affected by many variables, including pathogenic microbes, but it is unclear which microbes are most impactful and how they are transmitted into the eggs. This study characterized and compared the bacterial communities from the (i) cloaca of nesting sea turtles (ii) sand within and surrounding the nests; and (iii) hatched and unhatched eggshells from loggerhead (*Caretta caretta*) and green (*Chelonia mydas*) turtles. High throughput sequencing of bacterial 16S ribosomal RNA gene V4 region amplicons was performed on samples collected from 27 total nests in Fort Lauderdale and Hillsboro beaches in southeast Florida, United States. Significant differences were identified between hatched and unhatched egg microbiota with the differences caused predominately by *Pseudomona*s spp., found in higher abundances in unhatched eggs (19.29% relative abundance) than hatched eggs (1.10% relative abundance). Microbiota similarities indicate that the nest sand environment, particularly nest distance from dunes, played a larger role than the nesting mother's cloaca in influencing hatched and unhatched egg microbiota. Pathogenic bacteria potentially derive from mixed‐mode transmission or additional sources not included in this study as suggested by the high proportion (24%–48%) of unhatched egg microbiota derived from unknown sources. Nonetheless, the results suggest *Pseudomonas* as a candidate pathogen or opportunistic colonizer associated with sea turtle egg‐hatching failure.

## Graphical abstract

Here we used 16S rRNA gene sequencing to characterize and compare the bacterial communities of hatched and unhatched eggshells from loggerhead and green sea turtles to those in the cloaca of nesting sea turtles and sand within and surrounding the nests. Significant differences were identified between hatched and unhatched egg microbiota with the differences associated predominately with *Pseudomonas* spp., found in higher abundances in unhatched eggs than hatched eggs. Microbiota similarities indicate that the nest sand environment played a larger role than the nesting mother's cloaca in influencing egg microbiota. However, additional unknown sources may contribute to pathogenic bacteria introduction into unhatched eggs.

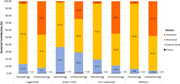

## 1 INTRODUCTION

Sea turtles (Order Testudines) have been considered iconic marine reptiles due to their graceful beauty, navigational prowess, size, and longevity. Once numbering in the millions according to voyage logs from the 1400 to 1600s (Jackson, 1997), sea turtle populations have dwindled to the thousands, and six of seven extant sea turtle species have been listed as endangered or threatened globally. Sea turtles are highly migratory marine reptiles who nest on tropical beaches and are found globally in temperate and tropical waters (Balazs, 1980; Snell & Fritts, 1983; Spotila, 2004). The reproductive strategy among the different species of sea turtles is similar; all are oviparous with high reproductive output to compensate for high natural mortality during early developmental stages (Butler, 1997; Wyneken et al., 1988). The eggs are fertilized internally, developing a soft shell before oviposition (Owens, 1980). After fertilization, females nest on sandy beaches depositing two to seven clutches of around 100 eggs per season (Carr, 1967). The eggs then incubate within the nest for 40–60 days, after which the hatchlings synchronize for a nighttime emergence (Carr & Hirth, 1961).

Survival rates of sea turtle eggs and hatchlings have decreased by different factors: physical nest destruction, predation, poaching, abiotic nest conditions (e.g., temperature, gas exchange, and moisture), and microbial interactions (Honarvar et al., 2011). Microbes live symbiotically with most eukaryotic organisms throughout their lifecycle (McFall‐Ngai et al., 2013), and this includes sea turtles from egg formation up to and throughout adulthood. The primary mechanisms for microbial introduction are hypothesized to be through either (i) maternal transmission during the two‐week formation period within the uterine tube and oviposition (Funkhouser & Bordenstein, 2013) or (ii) environmental transmission from sand surrounding the nest (Craven et al., 2007).

Maternal transmission is considered one of the main drivers of microbial introduction to sea turtle eggs. Sea turtle eggs spend the majority of their development (approximately two months) incubating in the sand without paternal care, therefore maternal transmission of bacteria can only occur during the two‐week formation period before oviposition (Miller, 1985). Sea turtle hatchlings have been found to acquire a portion of their normal flora from the mother before oviposition (Scheelings, 2019). The cloaca can potentially also introduce infectious microbes due to its function as a combined opening for the digestive, urinary, and reproductive systems in birds, reptiles, and amphibians. The turtle cloaca has been shown to hold a highly complex and variable microbiome (Al‐Bahry et al., 2009). Reproductive behaviors, environmental conditions, and diet can expose the cloaca to new and potentially pathogenic microbes. However, the establishment of new microbes in the cloaca depends on the presiding microbial community structure, the host's immune system, and the cloacal environment (Poiani, 2010). Additionally, mucus excreted during oviposition may contain antimicrobial properties that protect the embryos from potential pathogens in the environment or the reproductive tract (Keene, 2012; Praja et al., 2021; Soslau et al., 2011). This suggests that the transmission of microbes could occur at any time, whether it be before deposition (from the mother) or later on in the incubation process (from the environment).

Environmental transmission is considered another main driver of microbial introduction due to eggshell characteristics (Craven et al., 2007; De Reu et al., 2006). Sea turtles have a flexible eggshell composed of an inner organic membrane and an outer inorganic, calcareous layer that both contain numerous openings allowing for moisture and respiratory gas transfer (Al‐Bahry et al., 2009; Chan & Solomon, 1989). Environmental conditions in nests (e.g., temperature and oxygen content) have been found to correlate with the microbial assemblage present in the nest and with overall hatching success (Bézy et al., 2015). Physical properties of the nest substrate (e.g., sand grain size and organic matter content) establish diffusion rates of gases (e.g., oxygen and carbon dioxide), transport of water, and transmission of heat throughout the egg chamber, affecting developmental rates of the egg clutch (Ackerman, 1996; Mortimer, 1990). Biotic factors (e.g., clutch size and microbial activity) can alter nest temperature and gas composition, which can indirectly affect hatching success (Ackerman, 1996; Bézy et al., 2015; Mortimer, 1990; Prange & Ackerman, 1974).

Beach sands harbor diverse microbiomes that provide important ecosystem services, such as water purification, biogeochemical cycling, and organic compound mineralization (Boehm et al., 2014). Geographic and tidal zone locations, sand depth, water pollution, atmospheric deposition, and anthropogenic activity have been found to cause sand microbial variation between beaches (Boehm et al., 2014; Hu et al., 2019; Piggot et al., 2012; Staley & Sadowsky, 2016). Sand microbes from within sea turtle nests can also differ from the beach sand microbes due to the mucus excreted by the female providing a comparatively warm, moist environment and potential nutrient resource that promotes the growth of certain microbes within the egg chamber (Honarvar et al., 2011; Wyneken et al., 1988). When hatched and unhatched (also referred to as “failed”) egg microbiomes were previously characterized, differences in microbial species and overall microbial abundances were found to impact hatching success. Higher bacterial and fungal abundances and diversity in eggs (Gifari et al., 2018; Wyneken et al., 1988) and sand (Bézy et al., 2015; Honarvar et al., 2011) have been found to negatively impact the hatch rate success of a nest.

Research in sea turtle egg hatchability and their association with pathogens has been focused on sea turtle egg fusariosis (STEF) and *Fusarium solani* species complex (FSSC), which are fungal diseases causing high egg mortality in sea turtle nests worldwide (Gleason et al., 2020; Rosado‐Rodríguez & Maldonado‐Ramírez, 2016; Sarmiento‐Ramírez et al., 2010, 2014, 2017; Sidique et al., 2017; Smyth et al., 2019). STEF and FSSC are not the only fungi capable of penetrating and infecting sea turtle eggs. Additional fungal species identified in sea turtle eggs include *Aspergillus, Emericella, Rhizopus, Actinomucor*, and *Apophysomyces* (Candan, 2018). With research mostly focused on the fungal diseases impacting sea turtle hatching success, there is a lack of information on the role of bacteria within sea turtle eggs. Sarmiento‐Ramírez et al. (2014) provided the first analysis of bacterial composition in sea turtle eggs using PhyloChip methods. However, their study had a limited sampling size of four eggs (two hatched and two unhatched) collected from two nests and was focused on *Fusarium‐*infected nests. More research using new culture‐independent methods (e.g., massive parallel sequencing) and larger sampling efforts need to be conducted on uninfected nests to help determine the potential role of bacteria in sea turtle egg development.

To date, only a few studies have utilized high throughput sequencing (HTS) of metagenomic DNA to characterize sea turtle egg microbiota and correlate it with hatching success. Scheelings (2019) used HTS to determine how sea turtle hatchlings acquire their normal flora by comparing bacterial composition between freshly laid eggs and blood and gut samples from nesting mothers and hatchlings. Their results found that the eggs share 44% of their microbiota with the mother's blood, suggesting that a portion of their microbiota is transferred before shell formation (Scheelings, 2019). Hoh et al. (2020) used HTS to compare hatchery practices by the differences in bacterial and fungal pathogens found in the sand and *Fusarium*‐infected eggs. Their results found that eggs suffer an increased risk of infection in hatcheries that reuse sand for several nesting seasons (Hoh et al., 2020). Bézy et al. (2020) used HTS to compare the microbial composition in nest sand between areas of different embryo survivorship on a Costa Rica arribada beach but did not investigate the egg and cloaca microbiota. Their results found that sand microbial composition corresponds to particular environmental conditions suggesting that the presence of pathogenic microbes alone cannot fully determine hatching success (Bézy et al., 2020). Vecchioni et al. (2022) used HTS to characterize and compare loggerhead sea turtle egg microbiota along the Italian coasts of the Mediterranean Sea. Their results suggested that egg microbiota are shaped by maternal and environmental influences alongside a protective role of eggshells, but their study lacked microbial sampling from the nesting females to validate this claim (Vecchioni et al., 2022). Capri et al. (2023) used HTS and culture‐dependent methods to determine the bacteria and fungi responsible for hatching failure in two green sea turtle nests with different hatching success rates (0% and 59%). Their results found that differences in bacterial abundance may have a more predominant role in hatching success than fungi. Additionally, their results found that *Pseudomonas* and *Brucella* were the main bacteria affecting hatching success. Capri et al. (2023) also hypothesized that *Pseudomonas* derived from the sand while *Brucella* from the nesting female, however, their study lacks bacterial analysis from the nesting female to support this claim.

The primary aim of our study was to characterize the bacterial composition of hatched and unhatched eggs for two important turtle species, the loggerhead (*Caretta caretta*) and green (*Chelonia mydas*) turtles in southeast Florida, and compare them with samples taken from the cloaca of the nesting sea turtles and from the sand surrounding and within the nests. Identifying microbial differences between hatched and unhatched eggs from loggerhead and green turtles will lead to additional insights into the potential roles of bacteria in sea turtle eggs (e.g., commensals or pathogens). Comparisons between cloaca, sand, and egg samples will also provide new information on the possible transmission source for sea turtle egg bacterial microbiota.

## 2 EXPERIMENTAL PROCEDURES

### 2.1 Data acquisition

Sampling occurred during the 2021 nesting season (May–October) with help from the Broward County Sea Turtle Conservation Program (BCSTCP). Sampling was authorized by the Florida Fish and Wildlife Conservation Commission (FWC) under Marine Turtle Permit (MTP) #255. A total of 20 nests were sampled for loggerhead (*Caretta caretta*) and 7 nests for green (*Chelonia mydas*) turtles. The nests were determined when nesting turtles were identified by the BCSTCP staff under MTP #255 in Hillsboro and Fort Lauderdale beaches in Broward County, Florida. Broward County is located in southeast Florida and serves as a consistent annual nesting site for loggerheads (*C. caretta*), green turtles (*C. mydas*), and to a lesser extent leatherbacks (*Dermochelys coriacea*), which constitute approximately 90%–95%, 5%, and 1% of local nesting, respectively (Burkholder et al., 2020).

A total of nine different samples were collected from each nest (one cloaca, two sand, and six egg samples) resulting in 243 total samples being collected. Cloaca swabs were collected after oviposition while the turtle was temporarily detained by BCSTCP staff for research purposes (e.g., tagging and isotope and genetic sampling). A BD BBL™ CultureSwab™ EZ sterile media‐free polyurethane foam swab was dipped into sterile water and inserted into the cloaca (about 5–6 cm or no further than the point of resistance) where the epithelium was gently scraped for about 10 s. The remaining samples were collected following the nest excavation by the BCSTCP staff under MTP #214, which occurred three days after the nest hatch‐out had been documented following FWC Marine Turtle Guidelines. Two sand samples were collected from each nest using a sterile 2 mL microcentrifuge collection tube: the first (“nest sand”) was collected from the compact sand found at the bottom of the egg chamber, while the second (“control sand”) was collected from a human dug area of sand approximately 1–2 m away from the nest at the same depth as the egg chamber (range = 41–80 cm; Table 1). The latter sample was meant to represent sand samples with no association to any sea turtle nests (the original beach bacterial community). Within each nest, three hatched eggs and three unhatched eggs were swabbed on the interior portion of the eggshell for up to 30 s. Only hatched eggs that had more than 50% of the eggshell intact after hatchling emergence were selected since the interior could easily be distinguished from the exterior side. These hatched eggs also had a reduced chance of sand bacterial contamination affecting the eggshell's interior bacterial community due to the collapsing of the shell structure after turtle emergence. Only unhatched eggs that were not fully white, turgid, or demonstrated other characteristics suggestive of the presence of a living embryo were opened for swabbing. Eggs that were determined to be potentially viable were returned to the nest and reburied. All samples were placed on ice and transported to the laboratory to be stored at −80°C until DNA extractions began.

**Table 1:** Metadata associated with nests sampled.

Nest number	Turtle species	Beach	Latitude	Longitude	Bacterial incubation length (days)	Hatching success (%)	Clutch size	Chamber depth (cm)	High tide distance (m)	Dune distance (m)	Washover occurrences	pH (side, bottom)	Temperature (°C) (side, bottom)	Conductivity (µS/cm) (side, bottom)	Sand grain size (µm)	Sand sorting coefficient (S_0_)
127	Loggerhead	Hillsboro	26.266364	−80.079940	54	89.15	129	73	1.8	4.6	0	8.16, 8.13	31.8, 31.6	2, 52	442.984	1.286
236	Loggerhead	Hillsboro	26.271728	−80.079553	55	86.49	111	69	8.5	4.3	0	8.42, 7.22	33.9, 32.6	10, 66	399.827	1.269
242	Loggerhead	Hillsboro	26.301084	−80.076934	54	50.00	98	48	4.3	9.1	0	8.41, 8.04	31.9, 32.0	8, 35	344.769	1.315
282	Loggerhead	Hillsboro	26.273437	−80.079461	53	37.41	139	63	10.4	0.9	0	8.32, 7.57	33.5, 32.6	5, 20	465.131	1.445
322	Loggerhead	Hillsboro	26.281690	−80.078690	56	86.76	136	65	7.3	7.6	0	8.41, 8.24	32.5, 32.5	5, 35	414.71	1.276
323	Loggerhead	Hillsboro	26.291539	−80.077853	56	92.63	95	54	5.8	8.5	2	8.26, 8.36	32.2, 32.5	52, 21	368.333	1.282
392	Loggerhead	Hillsboro	26.282356	−80.078671	54	91.13	124	53	11.6	3.4	0	9.04, 8.37	32.9, 33.1	2, 36	369.319	1.277
395	Loggerhead	Hillsboro	26.294768	−80.077692	52	100.00	89	52	8.2	3.7	0	8.67, 8.41	33.5, 33.3	3, 36	349.142	1.296
456	Loggerhead	Fort Lauderdale	26.105561	−80.104279	55	92.62	122	54	8.5	80.5	0	8.50, 8.83	32.7, 33.0	3, 53	565.06	1.744
457	Loggerhead	Fort Lauderdale	26.103821	−80.104730	53	85.82	134	41	45.4	0.0	0	7.82, 8.45	32.4, 32.8	3, 131	450.679	1.556
463	Loggerhead	Hillsboro	26.288877	−80.078165	54	93.75	112	75	14.0	1.2	0	7.87, 8.08	31.9, 31.8	1, 16	538.46	1.662
463	Loggerhead	Fort Lauderdale	26.096287	−80.104877	55	95.58	113	63	12.2	110.0	1	8.16, 7.68	31.8, 32.2	91, 230	176.818	1.189
519	Loggerhead	Fort Lauderdale	26.099545	−80.104470	55	79.17	85	54	0.0	162.2	0	6.78, 6.78	32.0, 32.3	6, 70	580.429	1.844
520	Loggerhead	Fort Lauderdale	26.099477	−80.104516	53	89.90	99	49	3.4	154.5	0	6.78, 6.76	30.1, 31.3	7, 46	459.551	1.779
521	Loggerhead	Fort Lauderdale	26.097303	−80.104628	57	54.26	94	54	7.3	186.8	23	6.70, 6.73	29.7, 32.4	242, 2000	352.39	1.502
522	Loggerhead	Fort Lauderdale	26.095800	−80.104921	52	92.86	126	60	5.8	87.8	2	6.77, 6.82	29.7, 31.0	208, 133	473.56	1.836
697	Loggerhead	Fort Lauderdale	26.264892	−80.080085	50	95.05	101	51	4.6	95.4	3	6.79, 6.78	30.5, 30.8	351, 140	431.005	1.541
698	Loggerhead	Fort Lauderdale	26.266499	−80.079955	53	83.00	100	53	10.7	112.8	0	7.81, 7.83	32.4, 33.1	4, 48	394.668	1.382
699	Loggerhead	Fort Lauderdale	26.104836	−80.104836	51	79.07	129	48	15.8	159.7	0	8.17, 8.04	32.5, 32.7	6, 16	421.679	1.686
892	Loggerhead	Fort Lauderdale	26.102794	−80.104490	52	85.38	130	60	9.8	9.8	0	7.86, 7.85	29.5, 30.0	12, 70	425.691	1.248
695	Green	Hillsboro	26.099619	−80.104584	56	96.03	126	67	10.4	0.0	0	7.01, 6.92	30.5, 31.6	19, 236	377.553	1.273
696	Green	Hillsboro	26.168980	−80.097780	53	95.24	84	80	13.4	0.0	0	6.67, 6.77	33.3, 32.5	6, 59	477.532	1.551
823	Green	Fort Lauderdale	26.171648	−80.097618	52	92.56	121	68	13.4	6.4	0	6.41, 6.76	31.6, 31.4	3, 107	496.768	1.772
867	Green	Fort Lauderdale	26.153350	−80.100218	55	95.61	114	74	39.0	0.0	0	7.94, 7.93	28.6, 29.3	2, 113	457.801	1.549
1166	Green	Hillsboro	26.264360	−80.080120	57	97.43	116	77	11.6	0.0	0	7.6, 7.39	28.5, 29.2	2, 24	435.663	1.283
1167	Green	Hillsboro	26.269120	−80.079680	50	95.04	121	71	18.9	0.0	0	8.06, 8.12	31.0, 32.5	4, 67	409.351	1.300
1168	Green	Hillsboro	26.271610	−80.079620	55	95.69	116	60	11.3	0.0	0	8.08, 7.75	28.6, 29.5	16, 11	341.505	1.306

*Note*: Nest number indicates the nest number associated with the nest sampled on each beach, as determined by the BCSTCP. Turtle species (Loggerhead or Green Turtle), beach (Fort Lauderdale or Hillsboro), and GPS location (latitude and longitude) associated with each nest number are identified. Bacterial incubation length (days) was determined by the number of days between the date the nests were originally laid and the date of excavation. Hatching success was determined by dividing the number of hatched eggs (total number of hatched, LPIP, and DPIP eggs) by the total clutch size (total number of hatched, LPIP, DPIP, and whole eggs). Chamber depth was measured after all nest contents were removed, measuring from the bottom of the egg chamber to the topmost point of leveled sand. High tide and dune distance (m) were measured by BCSTCP staff when the nest was originally laid. Washover occurrences (50% of the nest was exposed to tidal action) were observed by BCSTCP staff throughout each nest's incubation period, total amounts of occurrences were calculated after nest removal. Conductivity, temperature, and pH were collected from the side and bottoms of each nest (both values shown in the table) immediately after nest contents were removed. Sand grain size and sorting coefficients were determined using 75 g of sand collected from within each egg chamber.

Abbreviations: BCSTCP, Broward County Sea Turtle Conservation Program; DPIP, dead pipped hatchlings; LPIP, live pipped hatchlings.

Environmental metadata were collected following standards set by Knight et al. (2012). At new nest sites, BCSTCP staff recorded the GPS location of the egg chamber, documented final nesting treatment (in situ [nest left where it was originally laid] or relocated [nest moved to a new location due to unsafe conditions]), and measured the nest's horizontal distance (m) from high tide and dunes using a Trimble GeoExplorer 6000 Series or Trimble GeoExplorer 2008 Series. Throughout the remainder of the incubation period, nest inundation and any instances of predation were recorded. Three days after the initial nest hatch‐out was marked by BCSTCP staff, the nest was excavated to determine hatching success through egg counts. The egg counts included the number of hatched eggs, live hatchlings in nest (LIN), dead hatchlings in nest (DIN), live pipped hatchlings (LPIP), dead pipped hatchlings (DPIP), and whole unhatched eggs. These were used to determine hatching success by dividing the number of hatched eggs (total number of hatched, LPIP, and DPIP eggs) by the total clutch size (total number of hatched, LPIP, DPIP, and whole eggs).

When all eggs were removed from the egg chamber, egg chamber depth (cm) was recorded. Nest sand pH (Hanna Instruments® HI981030 GroLine Soil pH Tester) and temperature and conductivity (Hanna Instruments® HI98331 Soil Test™) were measured at the bottom of the egg chamber and along the middle of the egg chamber wall to determine the differences experienced throughout the egg chamber. Sand grain size and sorting coefficient were determined by collecting 75 g of nest sand from each nest location into sterile 50 mL Falcon^TM^ centrifuge tubes. Sand samples were fractionated with a set of sieves (63, 125, 250, 500, 2000, and 4000 microns) to determine the particle‐size distribution by mass. The “G2Sd” R package (Fournier et al., 2014) was used to calculate the mean of the grain‐size distribution (geometric method of moments; Bunte & Abt, 2001) and sorting coefficient using the Trask Index (Trask, 1932) defined as D25/D75 (mm scale).

### 2.2 Bacterial sequencing

16S rRNA gene amplicons were sequenced using Earth Microbiome Project protocols for the Illumina MiSeq platform (Thompson et al., 2017). Microbial DNA was extracted from the cloaca, sand, and egg samples following protocols for the QIAGEN DNEasy PowerLyzer PowerSoil Kit. After extraction, each sample was checked using electrophoresis on a 1% agarose gel to ensure that the DNA was successfully extracted. Polymerase chain reaction (PCR) tests were run on the DNA isolates using Invitrogen™ Platinum™ Hot Start PCR Master Mix (2x). 515F and 806R primers were used to amplify the V4 region of the 16S rRNA gene (Caporaso et al., 2011; Easson & Lopez, 2019). The barcoded PCR products were then cleaned using Beckman Coulter AMPure XP beads, purifying the DNA amplicons away from contaminants such as dNTPs, salts, primers, and primer dimers (Greenwald et al., 2019). The final DNA concentration was checked using a Qubit® 2.0 Fluorometer and then diluted to a normalization of 4–5 ng/μL. Ten samples were then pooled at a time in equal volumes for a final quality check using Agilent 2200 and 4150 TapeStation Systems. A final library pool containing equal volumes of DNA products from all samples being sequenced was created and loaded into the Illumina MiSeq using the MiSeq Reagent Kit v3 at 600 cycles of sequencing following a modified Illumina workflow protocol used by the Microbiology and Genetics Laboratory at Nova Southeastern University Halmos College of Arts and Sciences.

### 2.3 Data analysis

The Quantitative Insights into Microbial Ecology v.2 (QIIME 2 v.2021.8) pipeline was used to perform microbial bioinformatics after sequencing was completed (Bolyen et al., 2019). Raw sequences were demultiplexed and quality‐filtered using DADA2 (Callahan et al., 2016). Amplicon sequence variants (ASV) were aligned with MAFFT (Katoh et al., 2002) and used to construct phylogeny with fasttree2 (Price et al., 2010). Taxonomy was assigned to the ASVs using the SILVA 138.1 feature classifier for the 515F/806R region of sequences (Quast et al., 2012; Yilmaz et al., 2014) after it was trained using scikit‐learn 0.24.1 (Bokulich et al., 2018). Before analysis, the feature table created by QIIME 2 was cleaned using the “decontam” (Davis et al., 2018) and “phyloseq” packages (McMurdie & Holmes, 2013) in R (v.4.0.2) (R Core Team, 2020). Two DNA extraction controls were sequenced and used as a reference for contamination cleaning using the prevalence method with a threshold of 0.1, which removed 21 ASVs. Further cleaning was done without the incorporation of DNA extraction controls using the frequency method with a threshold of 0.1, which removed an additional 107 ASVs. A final cleaning check was performed using the “vegan” package (Oksanen et al., 2020) where ASVs occurring less than 0.001% were removed, resulting in two additional ASVs being removed. Low threshold and abundance levels were used to preserve the bacterial diversity in the samples.

Alpha diversity, including Margalef's species richness (Margalef, 1958), Shannon diversity (Shannon & Weaver, 1949), and Inverse Simpson's diversity (Simpson, 1949), was assessed for each sample in the dataset. Generalized linear mixed‐effect models (GLMM) with gaussian distribution were performed using the “lme4” R package (Bates et al., 2015) for all samples to determine which variables affected each alpha diversity metric (response variables). Before setting up the model, the variables were assessed for covariation. Variables with strong correlations included latitude—longitude (corr = 0.996), latitude—dune distance (corr = −0.791), longitude—dune distance (corr = −0.757), temperature at bottom of nest—temperature at side of nest (corr = 0.873), and pH at bottom of nest—pH at side of nest (corr = 0.868). From these correlations, latitude, longitude, temperature at side of nest, and pH at side of nest were removed from the model while dune distance, temperature at bottom of nest, and pH at bottom of nest were retained. The fixed variables used within the “full” model were turtle species (categorical with two levels), beach (categorical with two levels), sample type (categorical with five levels), incubation length (continuous), clutch size (continuous), hatching success (continuous), chamber depth (continuous), high tide distance (continuous), dune distance (continuous), temperature at bottom of nest (continuous), pH at bottom of nest (continuous), conductivity at side of nest (continuous), conductivity at bottom of nest (continuous), sand grain size (continuous), and sorting coefficient (continuous). The interaction terms were sample type—turtle species and sample type—beach. Each model also included the nest number as a random effect to control for lack of independence between eggs from the same nest. Final “best‐fit” models were determined through term‐selection by AIC score comparisons. If the removal of a variable caused the AIC score to increase by a value of two or more than that variable was kept for the “best‐fit” model. The “best‐fit” models were validated by plotting Pearson residuals against fitted values, against each variable (covariate) in the model, and against each variable (covariate) not in the model. If the interaction terms were kept in the “best‐fit” model, then the “emmeans” package (Lenth, 2023) was used to perform pairwise analyses to determine which levels of the categorical variables had a significant effect.

To assess if sampling effort affected the differences in alpha diversity metrics between turtle species and beaches, sample‐size‐based rarefaction (interpolation) and prediction (extrapolation) curves were created using the “iNEXT” R package (Hsieh et al., 2016) by computing diversity estimates using the number of samples with respect to the total number of individuals (ASVs). These analyses (and future beta diversity analyses) were completed for each turtle species and beach separately. Comparisons between beaches were only made using loggerhead turtle samples, due to the low number of green turtle nests sampled at Fort Lauderdale (*n* = 2).

The data were then converted into relative abundance (resulting in proportional data for each sample) to perform beta diversity analyses between turtle species and beaches. Bray‐Curtis dissimilarity matrices with ASVs standardized by sample total were used to generate nonmetric multidimensional scaling plots using PRIMER v.7.0.17 software (Clarke & Gorley, 2015). One‐way, unordered analysis of similarity (ANOSIM; Clarke, 1993) with 9999 permutations was used to determine whether there were differences in community composition between sample types (hatched eggs, unhatched eggs, nest sand, control sand, and cloaca), beaches, and turtle species. Where significant differences were detected between groups, Similarity Percentages (SIMPER) analyses (Rees et al., 2004) with 9999 permutations were then used to determine which bacterial taxa were significantly different between sample types, beaches, and turtle species. Hatched and unhatched egg microbiota were compared to sand and cloaca microbiota to determine potential transmission sources using the “venn” R package (Dusa, 2021) to determine how many unique ASVs hatched and unhatched eggs had in common with cloaca and sand samples. SourceTracker (Knights et al., 2011) was then used to estimate the proportional contribution of each source type for hatched and unhatched egg microbiota using Bayesian modeling for proposed known (cloaca, control sand, and nest sand samples) and unknown source environments. An additional SourceTracker analysis was used to estimate the proportional contribution of control sand and cloaca microbiota on nest sand microbiota.

Differences between sample types were further visualized in R with the “phyloseq” and “microbiome” (Lahti & Shetty, 2019) packages using the relative abundances of the most abundant phyla and genera. Canonical analysis of principal coordinates (CAP; Anderson & Willis, 2003) was conducted in PRIMER v7 with PERMANOVA+ (Anderson et al., 2008) using Bray‐Curtis dissimilarity matrices analyzed against egg sample types. The CAP results were then overlayed with Pearson correlations to determine which bacteria best categorized and distinguished the hatched egg sample types from the unhatched egg sample types. To test if beach locale (GPS location) affected inter‐nest bacterial variance, Mantel tests were performed on control sand and nest sand microbiota (Bray‐Curtis distance matrices) with a matrix composed of straight‐line distances between nests. BIO‐ENV analyses (Clarke & Ainsworth, 1993) were conducted in “vegan” to correlate the relative abundance data (Bray‐Curtis dissimilarity with ASVs standardized by sample total) with the environmental data (Euclidean distances with scaled data) to determine what environmental factors best explained patterns of bacterial community change. Mantel tests (Mantel & Valand, 1970) with Spearman's rank correlations and 9999 permutations were performed after BIO‐ENV analyses to estimate significance.

## 3 RESULTS

### 3.1 Illumina MiSeq run and taxonomy statistics

Two Illumina MiSeq runs were used to sequence all 243 samples collected. The Q30 score for both runs was above 70% (77.21% and 70.42%) with more than 78% (85.63% and 78.9%) of clusters passing through Illumina's internal quality filtering procedure. On average, 117,698 (range: 46,217–237,925) high‐quality 16S rRNA gene sequences (V4 regions) were obtained from the 243 samples collected (Table A1). A total of 16,516 taxa were identified after data cleaning (the original raw count was 16,646 taxa). An average of 251 unique ASVs (range: 28–1644 ASVs) were identified per sample (Table A1).

### 3.2 Bacterial community compositions

**Figure 1:**
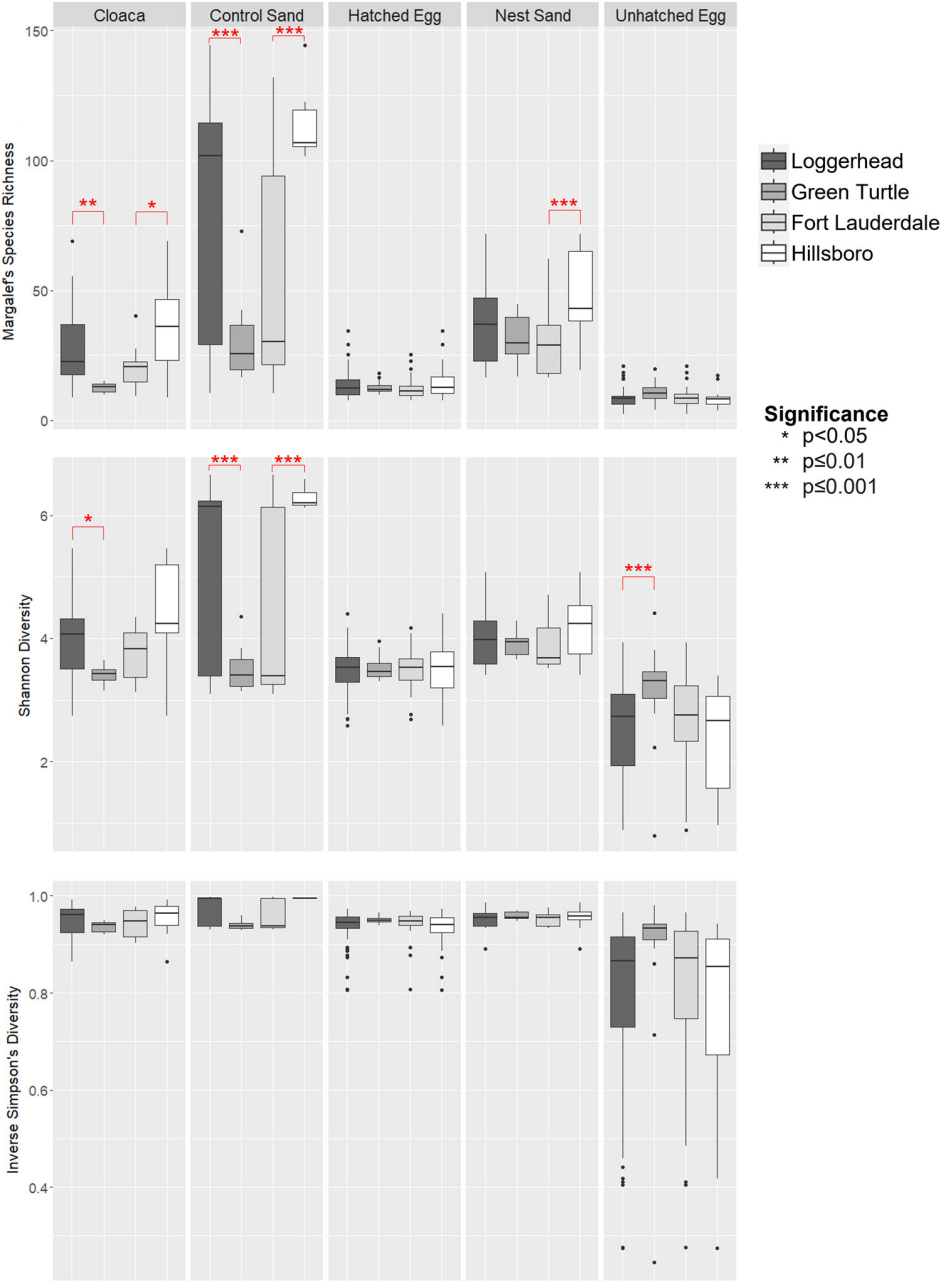
Alpha diversity comparison based on sample type. Three alpha diversity metrics were visualized: Margalef's species richness (top), Shannon diversity (middle), and Inverse Simpson's diversity (bottom). Facets were created by sample type (cloaca, control sand, hatched egg, nest sand, and unhatched egg) with each box within the facets representing a different dataset (loggerhead [dark grey], green turtle [grey], Fort Lauderdale [light grey], and Hillsboro [white]). The box portion shows the interquartile range separated by the median (black horizontal line) with whiskers (black vertical lines) showing the variability outside the upper and lower quartiles and outliers represented as black dots. Statistically significant differences, represented by the asterisks (*) were determined using the pairwise test in the “emmeans” package after the “best‐fit” model was determined (*p* values can additionally be found in Table A3). Cloaca samples were significantly different in bacterial richness and diversity between turtle species and were only significantly different between beaches (loggerhead samples only) for bacterial richness. Control sand samples were significantly different in bacterial richness and diversity between turtle species and beaches, while nest sand samples were only significantly different in bacterial richness between beaches. Unhatched eggs were significantly different in diversity between turtle species, while hatched eggs exhibited no significant differences.

**Figure 2:**
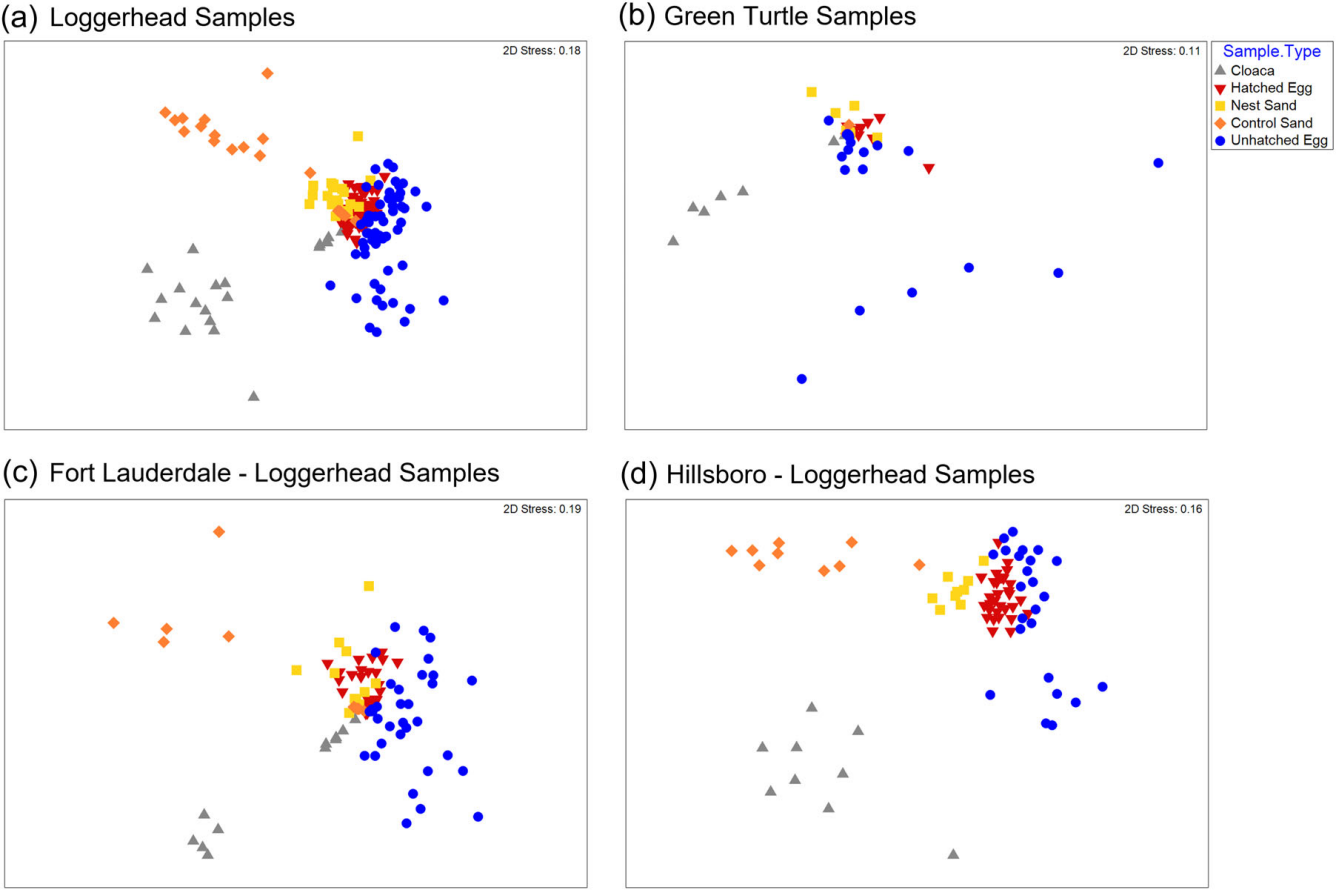
Bray‐Curtis dissimilarity nMDS comparisons of sample types in Loggerhead (a), Green Turtle (b), Fort Lauderdale (c), and Hillsboro (d) Samples. The similarity between sample types (cloaca [grey], control sand [orange], hatched egg [red], nest sand [gold], and unhatched egg [blue]) was visualized by nMDS plots in a two‐dimensional frame, using a Bray‐Curtis resemblance matrix to rank order the dissimilarity among pairs of samples. Stress values indicate the extent of the distortion to visualize the results in a two‐dimensional frame. All stress values were below 0.2, indicating a good representation of the data. Within the plots, the closer points (samples) are to each other, the more similar the community composition. In all plots, hatched eggs were closer to the nest sand samples, while the unhatched egg samples were closer to some of the cloaca samples instead of sand samples. Cloaca and control sand samples were separated further away from nest sand and egg samples. nMDS, nonmetric multidimensional scaling.

**Figure A1:**
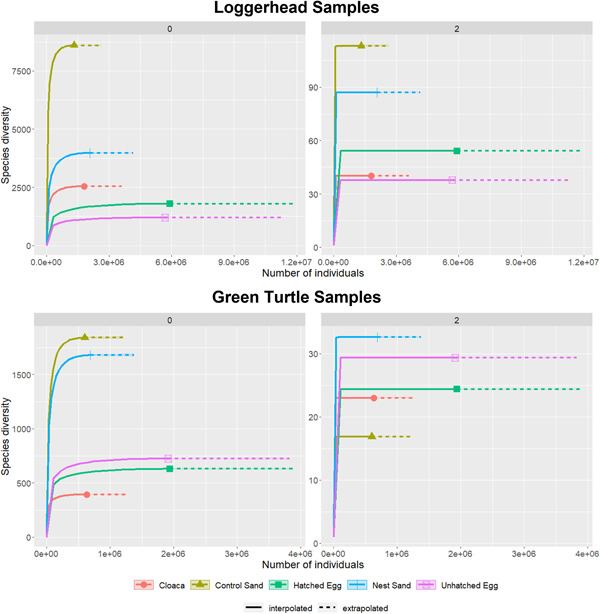
Sample‐sized‐based rarefaction (interpolation) and prediction (extrapolation) curves by sample type for each turtle species. Turtle species were analyzed separately: loggerhead (top plots) and green turtles (bottom plots). Plots on the left (*q* = 0) show bacterial taxa richness while plots on the right (*q* = 2) show Simpson diversity. Diversity was plotted against the number of ASVs (“individuals,” sample size) based on sample types: cloaca (red), control sand (brown), hatched egg (green), nest sand (blue), and unhatched egg (purple). The solid portion of the slopes represents the rarefaction (interpolation) of each sample type, while the dashed portion represents the predicted values (extrapolation) of each sample type. A higher diversity value at which the slope reaches a plateau indicates a sample type having greater richness or diversity. Loggerhead samples had greater bacterial richness and diversity than green turtle samples.

**Figure A2:**
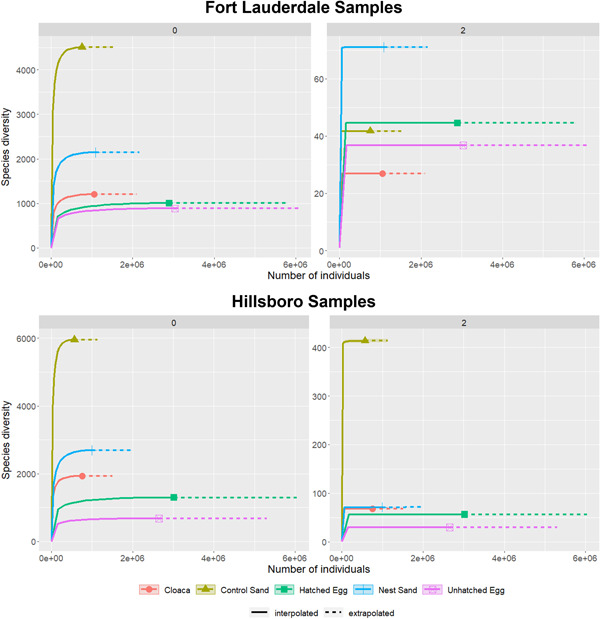
Sample‐sized‐based rarefaction (interpolation) and prediction (extrapolation) curves by sample type for each beach. Beaches were analyzed separately for loggerhead samples only: Fort Lauderdale (top plots) and Hillsboro (bottom plots). Plots on the left (*q* = 0) show bacterial taxa richness while plots on the right (*q* = 2) show Simpson diversity. Diversity was plotted against the number of ASVs (“individuals,” sample size) based on sample types: cloaca (red), control sand (brown), hatched egg (green), nest sand (blue), and unhatched egg (purple). The solid portion of the slopes represents the rarefaction (interpolation) of each sample type, while the dashed portion represents the predicted values (extrapolation) of each sample type. A higher diversity value at which the slope reaches a plateau indicates a sample type having greater richness or diversity. Hillsboro samples, except for unhatched eggs, had greater bacterial richness and diversity than Fort Lauderdale samples.

Control sand samples had significantly greater bacterial taxa richness and diversity when compared to egg and cloaca sample types in loggerhead and Hillsboro samples (Figure 1 and Table A2). Nest sand samples were significantly greater in bacterial taxa richness and diversity for both turtle species and beaches only when compared to hatched and unhatched eggs (Figure 1 and Table A2). Between egg sample types, hatched eggs had greater bacterial taxa richness and diversity than unhatched eggs (Figure 1 and Table A2). Loggerhead cloaca and control sand samples were significantly greater in bacterial richness and diversity than green turtles (Figure 1 and Table A3), while green turtle unhatched egg samples were significantly greater in diversity than loggerheads (Figure 1 and Table A3). Hillsboro control sand samples were significantly greater in bacterial richness and diversity than Fort Lauderdale (Figure 1 and Table A3). Although more samples were collected from loggerhead nests, these trends remained consistent when sample‐size was taken into consideration (Figures A1 and A2). One‐way ANOSIM tests showed that there were significant differences between the sample types in separate analyses for each turtle species (Loggerhead: *R* = 0.493, *p* < 0.001; Green Turtle: *R* = 0.154, *p* = 0.006) and each beach (Hillsboro: *R* = 0.659, *p* < 0.001; Fort Lauderdale: *R* = 0.339, *p* < 0.001). Nest sand microbiota were found to be more similar to hatched and unhatched egg microbiota than control sand and cloaca microbiota in both turtle species and beach comparisons (Figure 2 and Table A4). Interestingly, nest sand and control sand samples were found to be significantly different from one another in both turtle species and beach analyses (Figure 2 and Table A4).

### 3.3 Differences between hatched and unhatched egg microbiota

One‐way ANOSIM tests found significant differences in bacterial taxa between hatched and unhatched eggs in both turtle species (Loggerhead: *R* = 0.345, *p* < 0.001; Green: *R* = 0.132, *p* < 0.001) and beaches (Hillsboro: *R* = 0.421, *p* < 0.001; Fort Lauderdale: *R* = 0.262, *p* < 0.001). Loggerheads had a greater dissimilarity between hatched and unhatched egg microbiota than green turtles at the phylum (31.52% and 18.17%, respectively) and genus‐levels (77.00% and 43.68%, respectively) (Tables A5 and A6). Loggerhead hatched and unhatched egg microbiota were found to be more dissimilar within Hillsboro than Fort Lauderdale beaches at the phylum (34.20% and 29.01%, respectively) and genus‐levels (80.86% and 72.39%, respectively) (Tables A5 and A6).

**Figure 3:**
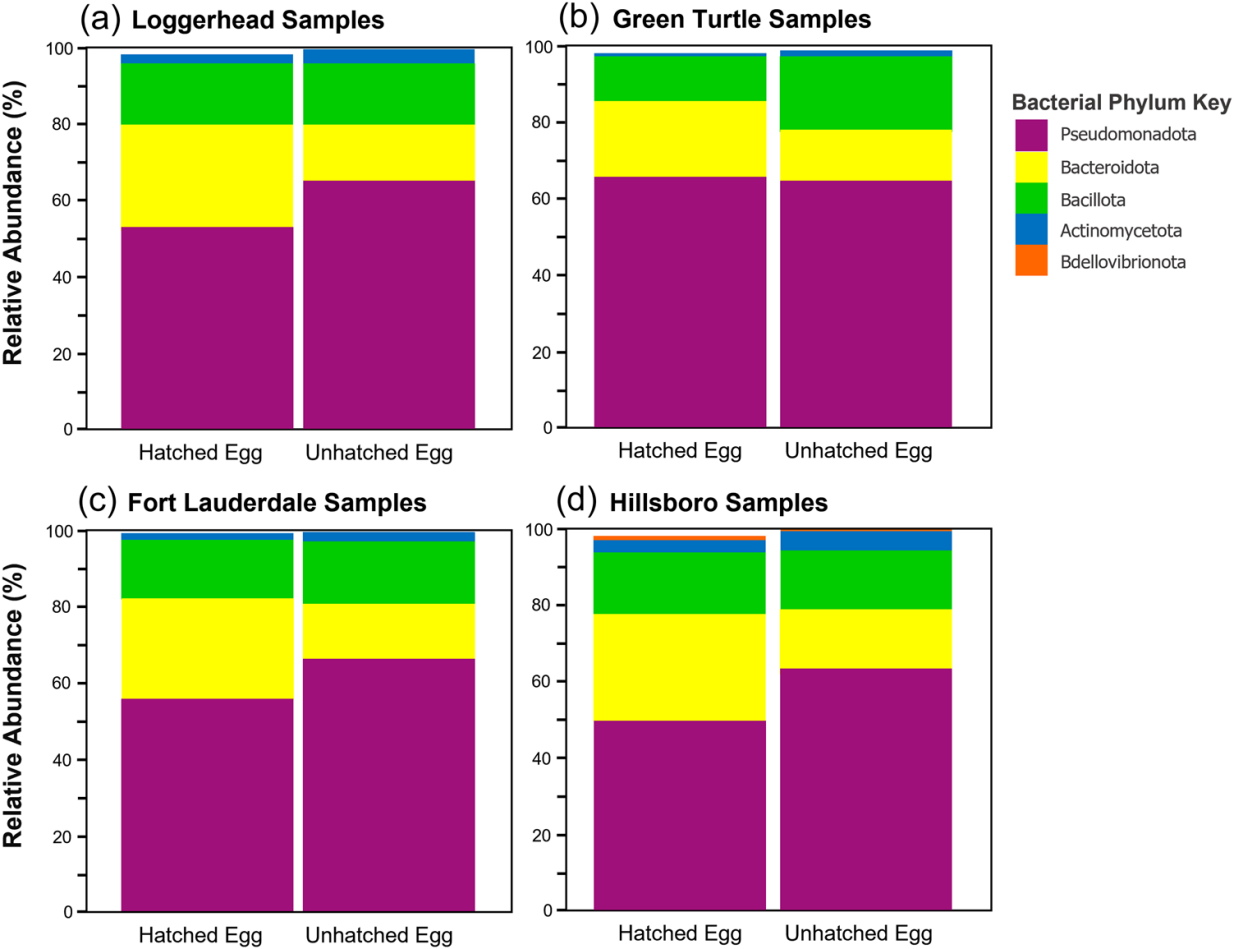
Most abundant bacterial phyla bar chart comparison of egg samples for turtle species and beaches. The most abundant bacterial phyla (based on relative abundance) were identified separately for each turtle species (Loggerhead [a] and Green Turtle [b]) and for the loggerhead samples at each beach (Fort Lauderdale [c] and Hillsboro [d]). Only bacterial phyla that had a relative abundance greater than 1% in either hatched or unhatched egg samples were selected for representation in these bar charts. All egg samples contained about 99% of the most abundant phyla identified. Loggerhead and both beach samples had a higher abundance of Pseudomonadota in unhatched egg samples. Both turtle species and Fort Lauderdale samples had a higher abundance of Bacillota in unhatched egg samples. Bacteroidota and Actinomycetota were in higher abundance in hatched eggs than unhatched eggs for both turtle species and beaches. Bdellovibrionota was only identified as an abundant bacterial phylum in Hillsboro samples and had a higher relative abundance in hatched eggs (1.01%) than unhatched eggs (0.04%).

**Figure 4:**
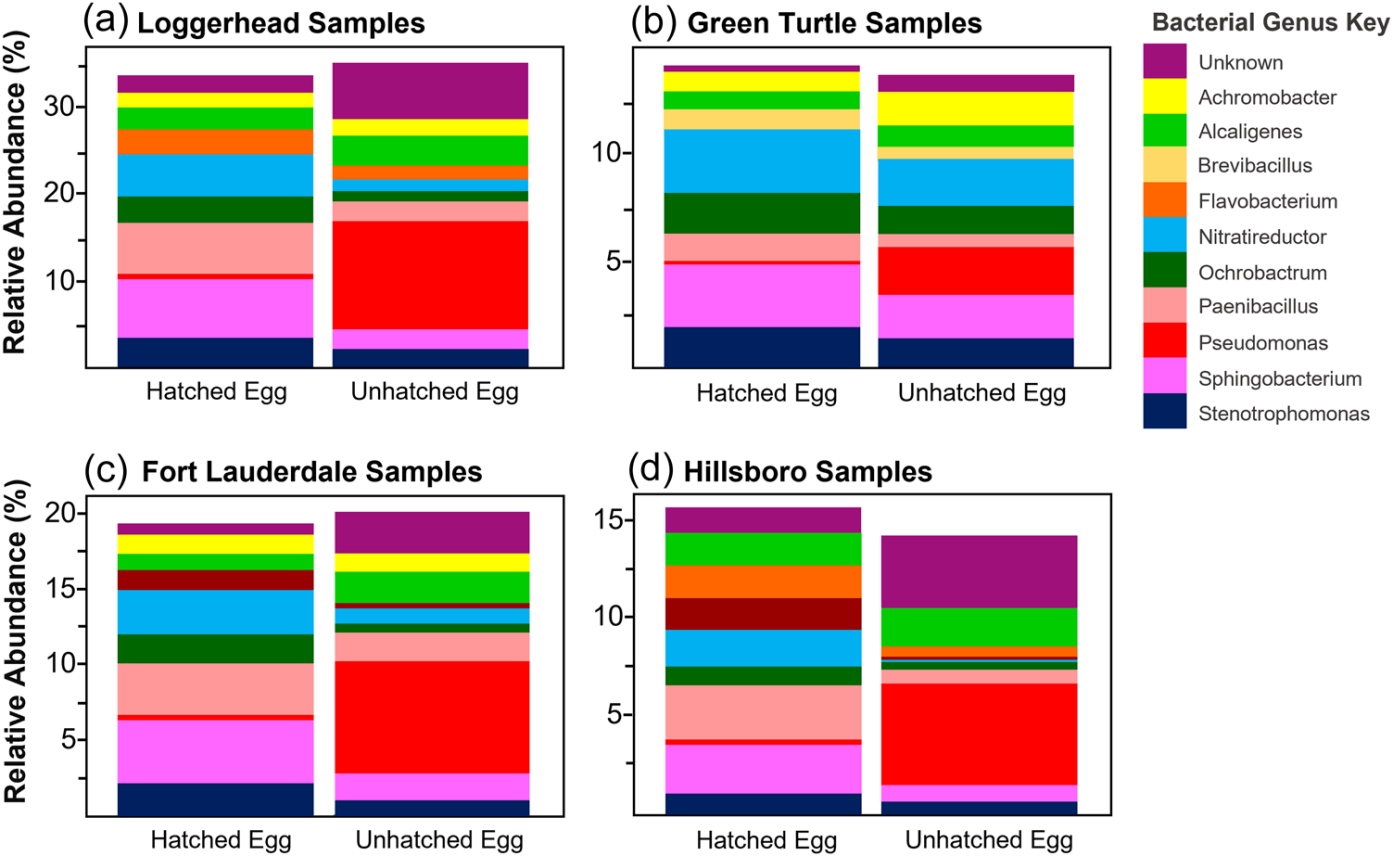
Most abundant bacterial genera bar chart comparison of egg samples for turtle species and beaches. The most abundant bacterial genera (based on relative abundance) were identified separately for each turtle species (Loggerhead [a] and Green Turtle [b]) and for the loggerhead samples at each beach (Fort Lauderdale [c] and Hillsboro [d]). Only bacterial genera that had a relative abundance greater than 1% in either hatched or unhatched egg samples were selected for representation in these bar charts. Loggerhead egg samples contained about 35% of the most abundant genera identified while green turtles were only composed of about 14%. Fort Lauderdale contained about 20% of the most abundant genera identified while Hillsboro was only composed of about 15%. Both turtle species and beaches had a higher abundance of *Pseudomonas* in unhatched egg samples while hatched eggs had higher abundances of *Sphingobacterium, Paenibacillus, Nitratireductor*, and *Lysobacter*.

Pseudomonadota (formerly known as Proteobacteria [Oren & Garrity, 2021]) was found as the highest correlated phylum to the differences between hatched and unhatched eggs for both turtle species and beaches and was found in higher abundances in unhatched eggs except in green turtle analyses (Figure 3 and Table A5). Actinomycetota (formerly known as Actinobacteria [Oren & Garrity, 2021]) and Bacillota (formerly known as Firmicutes [Oren & Garrity, 2021]) contributed higher abundances in unhatched eggs than hatched eggs (except for Bacillota in Hillsboro samples) while Bacteroidota contributed a higher abundance in hatched eggs than unhatched eggs (Figure 3 and Table A5). SIMPER analyses found *Pseudomonas* as the highest correlated genus to the differences between unhatched and hatched egg microbiota for both turtle species and beaches and was found in higher abundances in unhatched eggs (Figure 4 and Table A6). *Alcaligenes* also contributed a higher abundance in unhatched eggs than hatched eggs while *Nitratireductor, Flavobacterium, Paenibacillus*, and *Sphingobacterium* contributed higher abundances in hatched eggs (Figure 4 and Table A6).

Since hatched and unhatched eggs were found to be significantly different, canonical analysis of principal coordinates (CAP) was used to characterize the bacterial differences between the egg sample types. The CAP routine successfully separated egg samples with 93.827% (*m* = 39) correct classification. Only 1 out of 84 hatched eggs was misclassified, while 9 out of 78 unhatched eggs were misclassified. These CAP diagnostics indicate that the two sample types are distinct enough from one another to be used for predictive modeling and characterizing differences between egg‐hatching success. At the phylum level, hatched eggs were correlated (corr >0.3) with Bacteroidota, Verrucomicrobiota, Myxococcota, and Bdellovibrionota, while unhatched eggs were correlated with Pseudomonadota and Bacillota (Table A7). At the genus level, hatched eggs were correlated (corr >0.55) with *Sphingopyxis*, *Pseudonocardia*, *Devosia*, and *Cohnella*, while unhatched eggs were correlated with *Pseudomonas* (Table A7).

### 3.4 Potential transmission source

**Figure 5:**
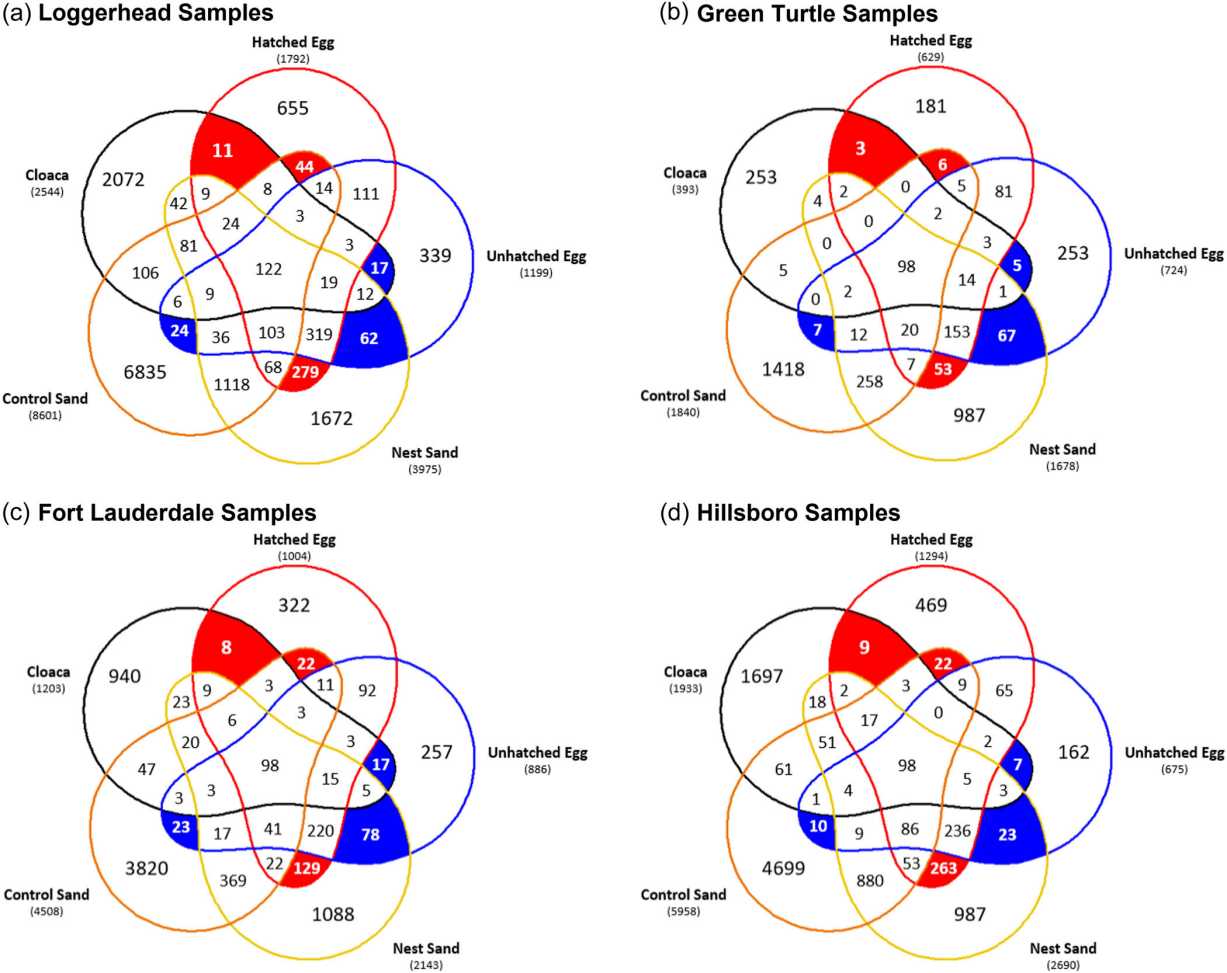
Unique ASV counts shared between sample types for Loggerhead (a), Green Turtle (b), Fort Lauderdale (c), and Hillsboro (d) samples. The number of shared ASVs between sample types (cloaca [black], control sand [orange], hatched egg [red], nest sand [gold], and unhatched egg [blue]) were identified separately for each turtle species (Loggerhead [a] and Green Turtle [b]) and for the loggerhead samples at each beach (Fort Lauderdale [c] and Hillsboro [d]). Unique ASVs hatched eggs shared with cloaca, control sand, and nest sand samples are highlighted in red, while unique ASVs unhatched eggs shared with cloaca, control sand, and nest sand samples are highlighted in blue. Highlighted values were divided by the total number of ASVs (in parentheses under sample type name) to determine the percentage of the total composition of the unique ASVs made up of hatched and unhatched egg samples. In each Venn diagram, both hatched and unhatched eggs shared more unique ASVs with nest sand samples than either the cloaca or control sand. ASV, amplicon sequence variant.

Cloaca, sand, and egg samples were compared to determine whether there was a greater maternal or environmental effect on egg microbiota. All egg samples shared more unique ASVs with nest sand samples (3%–20%) than the cloaca (0.5%–2%) or control sand (1%–2.6%) samples (Figure 5). Unhatched eggs shared more unique ASVs than hatched eggs with the cloaca samples (+0.21%–1.12%) while hatched eggs shared more with the control (+0.22%‐0.46%) and nest (+4.05%‐16.91%) sand samples (Figure 5). In green turtle samples, however, unhatched eggs shared more unique ASVs with all other sample types than hatched eggs (Figure 5). For both turtle species and beach comparisons, there were still 24%–37% unique ASVs within hatched and unhatched egg samples that did not match any ASVs found in the other sample types (Figure 5), indicating there may be another potential source for bacterial transmission. Most sediment habitats display some of the highest bacterial diversities, which is partially reflected by lower than expected sharing of ASVs between control sand and nest sand in loggerhead samples (Figure 2 and Table A4).

**Figure 6:**
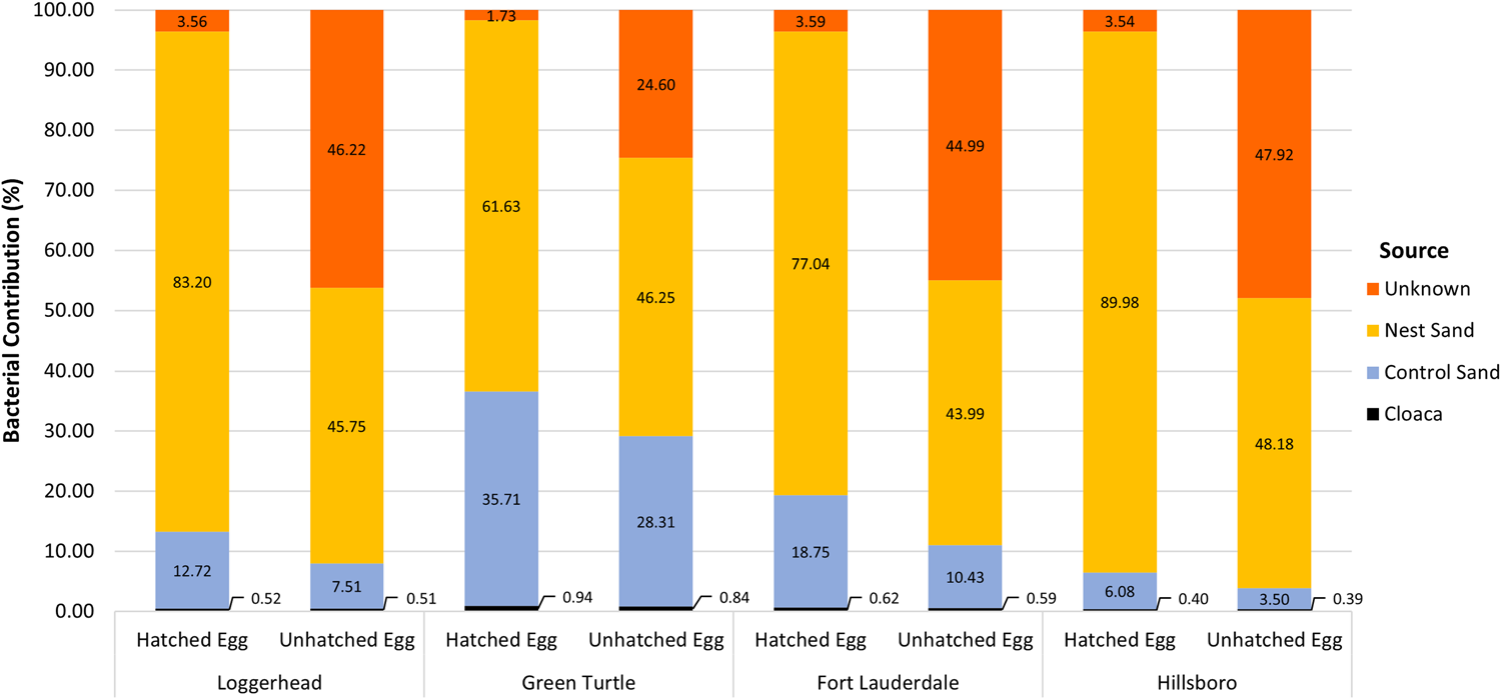
Source bacterial contributions for hatched and unhatched eggs. Proportional contributions of known (cloaca [black], control sand [blue], and nest sand [gold]) and unknown (orange) bacterial source environments were estimated in hatched and unhatched eggs for loggerheads, green turtles, Fort Lauderdale (loggerhead samples only), and Hillsboro (loggerhead samples only). The majority of hatched egg microbiota were derived from the nest sand. Unhatched egg microbiota had a greater derivation from unknown sources than hatched eggs.

SourceTracker analyses showed that nest sand most likely accounted for 61%–90% of bacterial introduction in hatched egg samples, identifying it as potentially the predominant source of bacterial communities (Figure 6). In unhatched egg samples, nest sand bacterial introduction only accounted for 44%–49% (Figure 6). A higher percentage of bacterial acquisition from an unknown source was identified in unhatched eggs (24%–48%) than hatched eggs (1%–4%; Figure 6). The cloaca was identified as the least likely origin of bacterial communities in sea turtle eggs, accounting for less than 1% of bacterial introduction in both egg sample types for all comparisons (Figure 6). The control sand was found to have a greater influence on green turtle egg microbiota (28%–36%) than loggerhead and beach comparisons (7%–13%; Figure 6). Since nest sand was identified as the predominant source of bacterial communities in egg samples, an additional SourceTracker analysis was run to determine if the nest sand had a stronger maternal (cloaca) or environmental (control sand) influence. Cloaca samples were found to account for only 0.89% of bacterial introduction in nest sand samples while control sand accounted for 46.55%, still, 52.56% of the nest sand bacterial source was identified from unknown sources.

Additional analyses were run to further corroborate that sand sources may influence bacterial transmission into sea turtle eggs more than cloaca microbiota. Although cloaca samples were found to be significantly different between turtle species (one‐way ANOSIM *R* = 0.561, *p* = 0.001), no significant differences in microbiota composition were found between turtle species for hatched or unhatched egg samples (one‐way ANOSIM; unhatched egg: *R* = −0.069, *p* = 0.886; hatched egg: *R* = −0.118, *p* = 0.979). On the contrary, control sand, nest sand, hatched egg, and unhatched egg microbiota showed to be significantly different between beaches (one‐way ANOSIM; nest sand: *R* = 0.158, *p* = 0.029; control sand: *R* = 0.274, *p* = 0.012; unhatched egg: *R* = 0.067, *p* = 0.032; hatched egg: *R* = 0.088, *p* = 0.002). A SIMPER analysis of the control sand showed that the main drivers of dissimilarity between beach microbiota were the genera *Achromobacter, Alcaligenes, Brevibacillus, Nitratireductor, Ochrobactrum, Sphingobacterium*, and *Stenotrophomonas* (Table A8).

### 3.5 Environmental factors shaping egg microbiota

Geographic location was first analyzed to determine if it played a stronger role in shaping sea turtle egg microbiota than the beach environment at each nest. Although there was a significant correlation between nest distances and control sand bacterial variance (Mantel *R* = 0.247, *p* = 0.018), no significant correlation was found between nest distances and nest sand bacterial variance (Mantel *R* = 0.071, *p* = 0.158). This suggests that although beach sand bacterial communities change with geographic location, more environmental factors play a role in shaping the different bacterial communities within the nest. Therefore, the different variations in environmental factors present at each nest may have stronger effects on shaping egg microbiota.

Three environmental variables were retained with all interactive variables in the GLMM analysis of Margalef's species richness, which strongly explained the variability in bacterial species richness (*R* ^2^ = 0.76; Table A9). Incubation length, high tide distance, and temperature at the bottom of nests were all found to positively affect bacterial species richness (*t* = 1.01, 3.13, and 1.48, respectively). Three environmental variables were retained with all interactive variables in the GLMM analysis of Shannon diversity, which strongly explained the variability in bacterial diversity (*R* ^2^ = 0.64; Table A9). Clutch size, high tide distance, and temperature at the bottom of nests were all found to positively affect bacterial diversity (*t* = 3.65, 2.01, and 1.66, respectively). One environmental variable was retained with only the sample type—beach interactive variable in the GLMM analysis of Inverse Simpson's diversity, which moderately explained the variability in bacterial diversity (*R* ^2^ = 0.25; Table A9). Sorting coefficient was found to negatively affect bacterial diversity (*t* = −0.24).

BIO‐ENV analyses showed that loggerhead egg samples had more environmental variables (six) explaining the change in bacterial communities when compared to green turtles (four) (Table A10). When analyzing beaches for loggerhead samples only, BIO‐ENV analyses showed that Hillsboro samples had more environmental variables (six) explaining the change in bacterial communities when compared to Fort Lauderdale (two) (Table A10). BIO‐ENV analyses determined that sand grain size for loggerhead and Fort Lauderdale samples (corr = 0.152 and 0.286, respectively), incubation length for green turtles (corr = 0.167), and conductivity at the side of nests (corr = 0.209) for Hillsboro samples were the single environmental variables which best explained correlation to bacterial community data.

Since significant differences were identified between hatched and unhatched eggs between beaches (and not between turtle species), a BIO‐ENV analysis was performed on the control sand samples to determine what environmental factors shaped the beach microbiota. Dune distance was identified as the best environmental factor to explain the bacterial differences between Fort Lauderdale and Hillsboro (corr = 0.252). Nests in Fort Lauderdale were found to occur significantly (*p* < 0.001) further from the dunes than nests in Hillsboro (average distance from dune = 89.7 m and 3.1 m, respectively, Table 1).

## 4 DISCUSSION

Our study presents the first in‐depth analysis of sea turtle egg bacterial communities in the continental United States using molecular methods and indicates that the nesting environment plays a critical role as a source of egg bacteria during the incubation period in loggerhead and green turtles in this region. The comparative approach with molecular methods has revealed several striking features of sea turtle egg and nest microbiota. First, hatched and unhatched eggs have statistically different bacterial assemblages from each other, with unhatched eggs having a more variable microbiota consistent with dysbiotic environments. Unhatched eggs represent the ultimate dysbiosis for a microsymbiont. Second, the nest sand microbiota have a greater influence on egg microbiota than cloaca microbiota. We hypothesize that this may be due to the waning influence of the mother's cloaca on the egg microbiota and/or the greater exposure to the nest sand during the two‐month incubation period before the eggs were sampled. Third, beach environments were found to significantly alter the egg microbiota, while turtle species had no statistically significant impact. Fourth, nest sand microbiota were distinct from control sand microbiota, potentially due to the introduction of the nesting female's mucus during oviposition.

### 4.1 Hatched and unhatched egg microbiota comparisons

Our study was able to collect samples from nests with hatching success rates ranging from 37% to 100% (Table 1) and detected significant microbiota composition differences between hatched and unhatched eggs. Hatched eggs were found to have less variance in their microbiota when compared to unhatched eggs (Figure 2). Less variance in hatched eggs may follow the so‐called “Anna Karenina principle” (AKP). The AKP posits that healthy animals possess relatively stable, similar microbiomes, while this stability can be disrupted by a variety of external stressors in unhealthy animals, resulting in more variable microbiomes (Zaneveld et al., 2017).

### 4.2 Hatched egg microbiota—The putative “healthy microbiota”

Based on the findings of the present study, hatched eggs correlated with having a lower species richness (between 300 and 400 ASVs) compared to unhatched eggs (>500 ASVs). At the phylum level, hatched (presumed healthy) eggs contained higher abundances of Bacteroidota, Verrucomicrobiota, Myxococcota, and Bdellovibrionota (Table A7). Bacteroidota and Verrucomicrobiota are phyla known for degrading complex organic matter, such as proteins and carbohydrates, which may provide the developing embryo with important nutritional requirements (He et al., 2017; Thomas et al., 2011). Bacteroidota has been found to possibly contribute to the formation of chicken embryonic intestinal microbiota during egg development (Ding et al., 2022), and may be expected to play a similar role in sea turtles. Myxococcota and Bdellovibrionota are phyla known for their predatory lifestyles. Myxococcota can secrete diverse secondary metabolites as antimicrobial proteins and metabolites, which are presumed to aid them in predating a broad range of microbes, including bacteria and fungi (Furness et al., 2020). Bdellovibrionota, on the other hand, is an obligate Gram‐negative predator (Li et al., 2021). In both cases, the higher abundance of predatory phyla may protect the turtle embryos from potential pathogens.

At the genus level, hatched eggs were correlated with higher abundances of *Sphingopyxis*, *Pseudonocardia*, *Devosia*, and *Cohnella* (Table A7). *Sphingopyxis* and *Devosia* are genera known for their degradation capabilities. *Sphingopyxis* have the potential to degrade a number of xenobiotics and other environmental contaminants, which helps them interact and survive in extreme environments (Sharma et al., 2021). Detoxification and degradation of organic pollutants have been identified as dominant functions of the genus *Devosia* (Talwar et al., 2020). The presence of bacteria capable of degrading environmental contaminants may protect the turtle embryos from being harmed during the incubation process. *Pseudonocardia* produce diverse secondary metabolites with antimicrobial bioactivities but are mostly known for their symbiotic relationship with fungus‐growing ants in which they inhibit entomopathogens that infect the ants (Goldstein & Klassen, 2020). The association with sea turtle eggs may play a role in protecting the eggs from fungal pathogens, such as *Fusarium* spp., which have been previously found in unhatched eggs (Brofft Bailey et al., 2018; Sarmiento‐Ramírez et al., 2010, 2014). *Cohnella* is a genus known for its cellulolytic or xylanolytic activities (Arneodo et al., 2019), whose relevance to sea turtle egg survival is yet to be determined. We posit that these compounds may be used to reduce root invasion into the eggshell from dune vegetation, which has been found to reduce hatching success in leatherback sea turtles (Conrad et al., 2011).

### 4.3 Unhatched egg microbiota—The putative “unhealthy microbiota”

Unhatched eggs were found to have a high species richness (>500 ASVs) within their microbiota, representing more variable bacterial assemblages than hatched eggs. At the phylum level, unhatched (presumed unhealthy) eggs were correlated with higher abundances of Pseudomonadota and Bacillota (Table A7). Pseudomonadota and Bacillota have previously been associated with disease suppression within the rhizosphere and have been previously identified in *Fusarium*‐infected sea turtle eggs (Mendes et al., 2011; Sarmiento‐Ramírez et al., 2014). The presence of certain Pseudomonadota (Betaproteobacteria, Gammaproteobacteria) has been suggested as a diagnostic for dysbiosis in humans due to their role in protein degradation and utilization of sugar and oxygen in the gut (Shin et al., 2015). Pseudomonadota has been previously suggested as an indicator of dysbiosis in sea turtles (Campos et al., 2018; Samuelson et al., 2020). Within the present study at the class level for Pseudomonadota, Gammaproteobacteria was found in higher abundance in unhatched eggs, while Alphaproteobacteria was found in higher abundance in hatched eggs (Table A7). Based on our results, higher relative abundances of Gammaproteobacteria could be indicative of dysbiosis within sea turtle egg microbiota. The ratio of Bacillota ("Firmicutes") to Bacteroidota ("Bacteroidetes") (F/B ratio) is widely used to track human intestinal homeostasis, with higher ratios being associated with dysbiosis (Abenavoli et al., 2019). The present study also showed a significant difference in the F/B ratio between hatched and unhatched eggs (two‐tailed paired samples Wilcoxon test: *p* = 0.006). Unhatched eggs were determined to have a significantly higher F/B ratio (average F/B ratio = 4.20) than hatched eggs (average F/B ratio = 0.71) (one‐tailed paired samples Wilcoxon test: *p* = 0.003). Further investigation into the F/B ratio in sea turtle eggs should be conducted as it could potentially be used to determine varying levels of dysbiosis within unhatched eggs.

At the genus level, unhatched eggs were correlated with higher abundances of *Pseudomonas* (Table A7). The genus *Pseudomonas* was identified as the major driver of the observed differences between hatched and unhatched egg samples, with a higher average relative abundance found in unhatched eggs (19.29%; variance = 7.34%) than in hatched eggs (1.10%; variance = 0.01%) (Figure 4 and Table A7). *Pseudomonas* spp. have been suggested to play a strong role in sea turtle egg hatching failure (Capri et al., 2023) and have been previously isolated and identified in unhatched sea turtle eggs from other studies (Awong‐Taylor et al., 2008; Craven et al., 2007; Keene, 2012; Wyneken et al., 1988). The genus *Pseudomonas* contains over 200 known taxa (Girard et al., 2021) that have high metabolic diversity, simple nutritional requirements, and wide temperature growth ranges (4°C–42°C) allowing them to inhabit a variety of environments (e.g., soil, water, plants, and association with larger organisms) as commensals and opportunistic pathogens (Chakravarty & Anderson, 2015). The diversity and adaptability of *Pseudomonas* may help explain why the taxon occurs widely in many environments and was found abundantly in unhatched eggs; however, we cannot be sure if the taxon caused the eggs to not hatch. *Pseudomonas* spp. have long been associated with proteinaceous food (i.e., eggs, milk, meat) spoilage under aerobic conditions (Raposo et al., 2016), so the presence of *Pseudomonas* may be due to the decomposition of the unhatched eggs. Future work could be done to try to establish the timeline in which *Pseudomonas* spp. enter the eggs to determine its functional role in sea turtle eggs.

*Alcaligenes* and *Achromobacter* were also found in higher abundance in unhatched eggs than hatched eggs (Figure 4). Both *Alcaligenes* and *Achromobacter* are known as opportunistic pathogens and are commonly found in soil, water, and intestinal tracts of vertebrates, such as loggerhead sea turtles (Busse & Stolz, 2006; Trotta et al., 2021). *Alcaligenes* spp. and *Achromobacter* spp. have recently been identified in sea turtle eggs, specifically green turtles (Candan & Candan, 2020), our study provides additional support to their presence and provides new insights into their higher abundances in unhatched eggs.

### 4.4 Beach environment influence and tracking potential transmission source

The transmission of pathogens into sea turtle eggs is unknown, but we can hypothesize that transmission occurs either through (i) maternal transmission during the two‐week formation period within the uterine tube and oviposition (Funkhouser & Bordenstein, 2013) or (ii) environmental transmission from sand surrounding the nest during the two‐month incubation period (Craven et al., 2007). Our study found that 44%–49% of unhatched eggs and 61%–90% of hatched egg microbiota derived from the nest sand microbiota, suggesting that the nest sand environment played a stronger role in shaping both egg microbiota than the cloaca, which was estimated to contribute less than 1% to both hatched and unhatched egg microbiota (Figure 6). Our results support the findings of Capri et al. (2023), who suggest that nest sand microbiota play a crucial role in shaping egg microbiota, with the different bacterial phyla present at each nest potentially being responsible for egg hatching failure. Due to the timing of egg sample collections (after incubation), we cannot dismiss maternal transfer as a critical component of sea turtle egg bacteria acquisition. Maternal transfer of bacteria may play a more important role during the initial stages of embryonic development within the female.

Maternal and environmental transmission, however, are not necessarily mutually exclusive. Sea turtle egg microbiota may potentially stem from a “starter” community from the nesting female, but over time, succession of environmental microbes may occur, depending on the original community composition established maternally, its resilience to change, and random, chance effects. Our study found that sea turtle nests host a different bacterial community than the original beach sand with only 46.55% of the nest microbiota being sourced from the control sand (Figure 2). We hypothesize that the distinction between the two communities may be a result of the mucus excreted by the female during oviposition. The potential antimicrobial properties, warm nest temperature (28.5°C–33.9°C in our study, Table 1), and added nutrients from the female mucus during oviposition may prime the nest sand and eggs to allow for the “starter” microbial community to colonize first during the initial stages of the incubation period. This would allow for a new microbial community to develop within the nest in place of the original beach microbial community. However, the cloaca was found to only account for 0.89% of bacterial introduction in nest sand samples suggesting that the change in nest sand composition from control sand cannot be directly attributed to the female cloaca. The “starter” microbial community within the nest from cloacal mucus may shift from the succession of environmental microbes over time due to the incubation length of sea turtle eggs (40–60 days).

There is also the potential for pathogens to be transmitted by multiple routes (Bright & Bulgheresi, 2010). The exact route of transmission for a pathogen may depend on the direct trade‐offs or indirect fitness effects caused by the different routes, the environment the pathogen is in, or based on symbiotic relationships with other microbes (Antonovics et al., 2017; Russell, 2019). Therefore, the likelihood of mixed‐mode transmission being used in sea turtle eggs appears high. For example, *Pseudomonas*, which was the most significant driver of differences between hatched and unhatched eggs, was present in all but three samples. Additionally, *Pseudomonas* spp. were found in low abundances in both cloaca (loggerhead = 0.21%, green turtle = 1.02%) and sand samples (loggerhead = 0.48% [control sand] and 1.32% [nest sand], green turtle = 0.81% [control sand] and 0.94% [nest sand]), making the exact route of transmission difficult to isolate. We hypothesize that pathogens may enter the eggs using a mixed‐mode route for sea turtle eggs due to the varying conditions of the female reproductive tract and final nesting locations.

We found that 28%–37% of hatched egg and 24%–35% of unhatched egg ASVs did not match the cloaca, control sand, or nest sand (Figure 5) and 1%–4% of hatched egg and 24%–48% of unhatched egg microbiota were determined to be from unknown sources (Figure 6). Therefore, there is still the potential for many other sources of transmission (e.g., water [rain or ocean], vegetation [dune or *Sargassum*], marine debris, and sand macroorganisms) due to the different location of each nest or additional sources before oviposition (e.g., paternal or other maternal sources [i.e., not the cloaca]). Different sea turtle nesting beaches could also be exposed to unique wildlife and vegetation or have different proximities to waste outflows and harbors that can impact nest microbiota.

We found that geographic location alone did not shape sea turtle egg microbiota, however, different combinations of environmental factors between turtle species and beaches explained the bacterial differences in egg microbiota (Tables A9 and A10). The variation in environmental factors between turtle species may be attributed to nest site selection differences between loggerhead and green turtles. Green turtle samples were found closer to dunes (average green turtle nest dune distance = 0.9 m [±2.2 m], loggerhead = 60.1 m [±66.0 m]), while loggerhead samples were closer to the high tide line (average loggerhead nest high tide distance = 9.8 m [±9.1 m], green turtle = 16.9 m [±10.2 m]; Table 1).

Beach composition, specifically beach width, may also have affected nest site selection and may also explain the variation in environmental factors between beaches. Hillsboro beaches are known for having narrower littoral zones thus restricting the dispersal of microbes and having a greater amount of vegetation and less human activity due to it being a residential beach whereas Fort Lauderdale is more commercialized with wider beaches and less vegetation. Nests were laid further from both the high tide line and dunes in Fort Lauderdale (average distance from high tide line = 13.5 m [±13.0 m], dune = 89.7 m [±64.9 m]) than in Hillsboro (average distance from high tide line = 9.8 m [±4.2 m], dune = 3.1 m [±3.3 m]; Table 1). Dune distance was found to play the most significant role in shaping the bacterial differences in beach sand composition in BIO‐ENV analyses. Nest distance from the high tide line was potentially not isolated as the best environmental factor in the BIO‐ENV analysis because the tidal distance is constantly changing, while the dune distance is a more permanent variable. However, high tide distance was found to significantly affect the bacterial richness and Shannon diversity in the GLMM analyses (Table A9).

Our hypothesis that the local beach environment has the largest effect in shaping sea turtle egg microbiota rather than the nesting mother's microbiota counters previous research that vertical (parent‐offspring) symbiont transmission increases in terrestrial environments and decreases in aquatic environments (Russell, 2019). Sea turtle nests primarily occur in littoral zones (the average distances of all 2021 sampled nests from the high tide line were 11.6 m [±9.7 m] and 44.8 m [±62.5 m] from the dunes; Table 1); thus, the nests have the opportunity to be exposed to periodic tidal washover and aquatic microbiota. Horizontal (environment‐offspring) transmission of aquatic bacteria may increase with nests that are near the high tide line due to increased washover occurrences or from the introduction of pathogens from terrestrial run‐off. Nests that are laid near the high tide line have the potential to also be introduced to high levels of fecal indicator bacteria, which have been found to exceed set colony‐forming unit levels 2.475 times more during high tide than low tide on South Florida beaches (Aranda et al., 2016). The beach environment, particularly dune distance, was found to play a role in shaping the bacterial community differences within control sand samples. Similarly, nest sand communities can potentially be affected by the beach environment and cause egg bacterial differences, such as species richness and diversity, as we saw in our analyses (Table A9).

Differences in control sand microbiota between beaches were found to be mainly caused by the genera *Sphingobacterium*, *Ochrobactrum, Achromobacter*, and *Nitratireductor*, which all occurred in higher abundances in Fort Lauderdale than Hillsboro. Additionally, *Sphingobacterium*, *Ochrobactrum*, and *Nitratireductor* were all found in higher abundances in hatched eggs than unhatched eggs on both beaches (Figure 4). This may explain why Fort Lauderdale had a significantly higher average hatching success rate (88.78%) when compared to Hillsboro (82.62%) overall in the 2021 nesting season for loggerheads (nonparametric two‐tailed *t*‐test: *W* = 323344, *p* < 0.001). Our findings suggest that the localized environmental conditions and bacteria at each nest may play an important role in shaping the egg microbiota and potentially affecting their hatching success, as previously suggested (Capri et al., 2023). Further research should be conducted to understand the role these bacteria play in egg‐hatching success and their use in beach ecosystem dynamics.

## 5 CONCLUSIONS

Studying sea turtle egg microbiomes and their transmission can aid in better conservation methods and management that will then aid in population recovery. Our study produced new insights into the bacterial communities associated with hatched and unhatched eggs in loggerhead and green turtle nests and was the first to find that the environment plays a stronger role in shaping sea turtle egg microbiota than the nesting female. Future research should be conducted to further corroborate these results and determine ways to monitor important sea turtle nesting locations for potentially pathogenic bacteria. Additionally, similar research can be conducted on cloaca, sand, and egg (unhatched and hatched) samples but with the inclusion of mycobiome analyses with the current bacterial analyses. By not including the mycobiome in our current study, a key element of variation in microbiome‐associated hatchability (e.g., *Pseudomonas* associated with fungal growth in unhatched eggs) could be missing and should be further investigated.

Since we found that nest sand is a main source of bacterial transmission for sea turtle eggs, further research should be conducted where the nest's microbiota and environmental parameters (e.g., temperature, gas diffusion and presence, and moisture levels) are analyzed continuously throughout the eggs' incubation period. This would provide better insight into what environmental parameters cause the inundation of pathogens and see if there is a particular stage during the incubation period that it occurs. Additionally, mechanical beach cleaning and beach renourishment projects should be monitored on critical sea turtle nesting beaches to determine if these human activities are altering the sea turtle nesting microbiota and bacterial loads.

Knowing which bacteria are potentially pathogenic to sea turtle eggs could provide additional details to allow for targeted beach monitoring to promote greater hatching success for loggerhead and green turtles in South Florida. This information can also be used to help mitigate human impacts on pathogen transmission into sea turtle eggs. Ideal sea turtle nest relocation zones can be identified on important sea turtle nesting beaches by analyzing the sand microbiota for bacteria correlated with higher hatching success rates and healthy sea turtle eggs. Additionally, sand that is being used for beach renourishment projects can be analyzed for any potentially harmful bacteria before placement in important sea turtle nesting areas to reduce the chance of introducing pathogenic bacteria to sea turtle nests. Sea turtle conservation depends on healthy nesting and hatching, identifying the bacteria influencing the success of sea turtle eggs and understanding their transmission can help reduce threats to their conservation and help protect and preserve these endangered species.

## AUTHOR CONTRIBUTIONS

**Colleen M. McMaken**: data curation (lead), formal analysis (lead), funding acquisition (equal), methodology (lead), visualization (lead), writing—original draft (lead), writing—review & editing (equal); **Derek A. Burkholder**: conceptualization (equal), investigation (supporting), methodology (supporting), writing—review & editing (equal); **Rosanna J. Milligan**: data curation (supporting), formal analysis (supporting), visualization (supporting), writing— review & editing (equal); **Jose V. Lopez**: conceptualization (equal), data curation (supporting), formal analysis (supporting), funding acquisition (equal), investigation (supporting), methodology (supporting), writing—review & editing (equal).

## CONFLICT OF INTEREST STATEMENT

The authors declare no conflicts of interest.

## ETHICS STATEMENT

All prevailing local, national, and international regulations and conventions, and normal scientific ethical practices, have been respected. Sampling was authorized by the Florida Fish and Wildlife Conservation Commission (FWC) under Marine Turtle Permit (MTP) #255.

## Data Availability

Final sequence data were uploaded to NCBI's Short Read Archive (BioProject accession: PRJNA804903: https://www.ncbi.nlm.nih.gov/bioproject/PRJNA804903. Full data files (lists for potential pathogens, shared and unique ASVs, SIMPER analyses, Krona plots) along with the R scripts and files used for this analysis can be found at https://github.com/colmcmaken/Turtle-Egg-Microbiome.

## 1

**Table A1:** Number of sequence reads and bacterial taxonomy count per sample.

Sample ID	Nest number	Sample type	Turtle species	Beach	QIIME sequence reads	ASV count
C.1166.CM.H	1166	Cloaca	Green Turtle	Hillsboro	100279	121
C.1167.CM.H	1167	Cloaca	Green Turtle	Hillsboro	127488	113
C.1168.CM.H	1168	Cloaca	Green Turtle	Hillsboro	170528	153
C.127.CC.H	127	Cloaca	Loggerhead	Hillsboro	46217	268
C.236.CC.H	236	Cloaca	Loggerhead	Hillsboro	123741	797
C.242.CC.H	242	Cloaca	Loggerhead	Hillsboro	59700	98
C.282.CC.H	282	Cloaca	Loggerhead	Hillsboro	61555	506
C.322.CC.H	322	Cloaca	Loggerhead	Hillsboro	103713	631
C.323.CC.H	323	Cloaca	Loggerhead	Hillsboro	125246	457
C.392.CC.H	392	Cloaca	Loggerhead	Hillsboro	137084	424
C.395.CC.H	395	Cloaca	Loggerhead	Hillsboro	90439	204
C.456.CC.F	456	Cloaca	Loggerhead	Fort Lauderdale	143942	261
C.457.CC.F	457	Cloaca	Loggerhead	Fort Lauderdale	84848	451
C.463.CC.F	463	Cloaca	Loggerhead	Fort Lauderdale	114538	317
C.463.CC.H	463	Cloaca	Loggerhead	Hillsboro	140709	270
C.519.CC.F	519	Cloaca	Loggerhead	Fort Lauderdale	135444	243
C.520.CC.F	520	Cloaca	Loggerhead	Fort Lauderdale	51276	101
C.521.CC.F	521	Cloaca	Loggerhead	Fort Lauderdale	146274	139
C.522.CC.F	522	Cloaca	Loggerhead	Fort Lauderdale	135869	164
C.695.CM.H	695	Cloaca	Green Turtle	Hillsboro	113633	130
C.696.CM.H	696	Cloaca	Green Turtle	Hillsboro	83683	170
C.697.CC.F	697	Cloaca	Loggerhead	Fort Lauderdale	113112	242
C.698.CC.F	698	Cloaca	Loggerhead	Fort Lauderdale	132994	259
C.699.CC.F	699	Cloaca	Loggerhead	Fort Lauderdale	136480	185
C.823.CM.F	823	Cloaca	Green Turtle	Fort Lauderdale	115898	167
C.867.CM.F	867	Cloaca	Green Turtle	Fort Lauderdale	130248	155
C.892.CC.F	892	Cloaca	Loggerhead	Fort Lauderdale	165918	227
H1.1166.CM.H	1166	Hatched Egg	Green Turtle	Hillsboro	126697	135
H1.1167.CM.H	1167	Hatched Egg	Green Turtle	Hillsboro	148181	192
H1.1168.CM.H	1168	Hatched Egg	Green Turtle	Hillsboro	170681	152
H1.127.CC.H	127	Hatched Egg	Loggerhead	Hillsboro	65207	157
H1.236.CC.H	236	Hatched Egg	Loggerhead	Hillsboro	125762	137
H1.242.CC.H	242	Hatched Egg	Loggerhead	Hillsboro	115605	188
H1.282.CC.H	282	Hatched Egg	Loggerhead	Hillsboro	107474	244
H1.322.CC.H	322	Hatched Egg	Loggerhead	Hillsboro	115932	151
H1.323.CC.H	323	Hatched Egg	Loggerhead	Hillsboro	71369	93
H1.392.CC.H	392	Hatched Egg	Loggerhead	Hillsboro	114411	113
H1.395.CC.H	395	Hatched Egg	Loggerhead	Hillsboro	102350	104
H1.456.CC.F	456	Hatched Egg	Loggerhead	Fort Lauderdale	127637	129
H1.457.CC.F	457	Hatched Egg	Loggerhead	Fort Lauderdale	126113	297
H1.463.CC.F	463	Hatched Egg	Loggerhead	Fort Lauderdale	70929	92
H1.463.CC.H	463	Hatched Egg	Loggerhead	Hillsboro	149186	265
H1.519.CC.F	519	Hatched Egg	Loggerhead	Fort Lauderdale	137968	122
H1.520.CC.F	520	Hatched Egg	Loggerhead	Fort Lauderdale	85425	87
H1.521.CC.F	521	Hatched Egg	Loggerhead	Fort Lauderdale	77047	88
H1.522.CC.F	522	Hatched Egg	Loggerhead	Fort Lauderdale	121755	136
H1.695.CM.H	695	Hatched Egg	Green Turtle	Hillsboro	113254	140
H1.696.CM.H	696	Hatched Egg	Green Turtle	Hillsboro	87856	150
H1.697.CC.F	697	Hatched Egg	Loggerhead	Fort Lauderdale	106900	109
H1.698.CC.F	698	Hatched Egg	Loggerhead	Fort Lauderdale	111885	143
H1.699.CC.F	699	Hatched Egg	Loggerhead	Fort Lauderdale	88232	109
H1.823.CM.F	823	Hatched Egg	Green Turtle	Fort Lauderdale	146423	118
H1.867.CM.F	867	Hatched Egg	Green Turtle	Fort Lauderdale	139709	132
H1.892.CC.F	892	Hatched Egg	Loggerhead	Fort Lauderdale	109597	137
H2.1166.CM.H	1166	Hatched Egg	Green Turtle	Hillsboro	119057	131
H2.1167.CM.H	1167	Hatched Egg	Green Turtle	Hillsboro	122004	207
H2.1168.CM.H	1168	Hatched Egg	Green Turtle	Hillsboro	160511	156
H2.127.CC.H	127	Hatched Egg	Loggerhead	Hillsboro	81923	190
H2.236.CC.H	236	Hatched Egg	Loggerhead	Hillsboro	99275	193
H2.242.CC.H	242	Hatched Egg	Loggerhead	Hillsboro	83242	140
H2.282.CC.H	282	Hatched Egg	Loggerhead	Hillsboro	125318	341
H2.322.CC.H	322	Hatched Egg	Loggerhead	Hillsboro	139238	135
H2.323.CC.H	323	Hatched Egg	Loggerhead	Hillsboro	93569	133
H2.392.CC.H	392	Hatched Egg	Loggerhead	Hillsboro	98448	145
H2.395.CC.H	395	Hatched Egg	Loggerhead	Hillsboro	103014	94
H2.456.CC.F	456	Hatched Egg	Loggerhead	Fort Lauderdale	140092	231
H2.457.CC.F	457	Hatched Egg	Loggerhead	Fort Lauderdale	102299	263
H2.463.CC.F	463	Hatched Egg	Loggerhead	Fort Lauderdale	105816	154
H2.463.CC.H	463	Hatched Egg	Loggerhead	Hillsboro	190432	200
H2.519.CC.F	519	Hatched Egg	Loggerhead	Fort Lauderdale	100963	179
H2.520.CC.F	520	Hatched Egg	Loggerhead	Fort Lauderdale	73146	85
H2.521.CC.F	521	Hatched Egg	Loggerhead	Fort Lauderdale	91415	137
H2.522.CC.F	522	Hatched Egg	Loggerhead	Fort Lauderdale	130659	144
H2.695.CM.H	695	Hatched Egg	Green Turtle	Hillsboro	96809	130
H2.696.CM.H	696	Hatched Egg	Green Turtle	Hillsboro	105326	168
H2.697.CC.F	697	Hatched Egg	Loggerhead	Fort Lauderdale	123403	144
H2.698.CC.F	698	Hatched Egg	Loggerhead	Fort Lauderdale	137664	146
H2.699.CC.F	699	Hatched Egg	Loggerhead	Fort Lauderdale	109190	115
H2.823.CM.F	823	Hatched Egg	Green Turtle	Fort Lauderdale	162447	116
H2.867.CM.F	867	Hatched Egg	Green Turtle	Fort Lauderdale	140323	131
H2.892.CC.F	892	Hatched Egg	Loggerhead	Fort Lauderdale	126565	153
H3.1166.CM.H	1166	Hatched Egg	Green Turtle	Hillsboro	113366	131
H3.1167.CM.H	1167	Hatched Egg	Green Turtle	Hillsboro	129611	180
H3.1168.CM.H	1168	Hatched Egg	Green Turtle	Hillsboro	119282	133
H3.127.CC.H	127	Hatched Egg	Loggerhead	Hillsboro	105554	178
H3.236.CC.H	236	Hatched Egg	Loggerhead	Hillsboro	96562	267
H3.242.CC.H	242	Hatched Egg	Loggerhead	Hillsboro	69680	110
H3.282.CC.H	282	Hatched Egg	Loggerhead	Hillsboro	163402	409
H3.322.CC.H	322	Hatched Egg	Loggerhead	Hillsboro	113001	145
H3.323.CC.H	323	Hatched Egg	Loggerhead	Hillsboro	92665	190
H3.392.CC.H	392	Hatched Egg	Loggerhead	Hillsboro	151009	200
H3.395.CC.H	395	Hatched Egg	Loggerhead	Hillsboro	103384	95
H3.456.CC.F	456	Hatched Egg	Loggerhead	Fort Lauderdale	120150	174
H3.457.CC.F	457	Hatched Egg	Loggerhead	Fort Lauderdale	111353	212
H3.463.CC.F	463	Hatched Egg	Loggerhead	Fort Lauderdale	110824	104
H3.463.CC.H	463	Hatched Egg	Loggerhead	Hillsboro	220501	141
H3.519.CC.F	519	Hatched Egg	Loggerhead	Fort Lauderdale	101976	112
H3.520.CC.F	520	Hatched Egg	Loggerhead	Fort Lauderdale	83727	98
H3.521.CC.F	521	Hatched Egg	Loggerhead	Fort Lauderdale	122011	130
H3.522.CC.F	522	Hatched Egg	Loggerhead	Fort Lauderdale	98815	120
H3.695.CM.H	695	Hatched Egg	Green Turtle	Hillsboro	96778	148
H3.696.CM.H	696	Hatched Egg	Green Turtle	Hillsboro	102833	151
H3.697.CC.F	697	Hatched Egg	Loggerhead	Fort Lauderdale	95256	118
H3.698.CC.F	698	Hatched Egg	Loggerhead	Fort Lauderdale	150883	123
H3.699.CC.F	699	Hatched Egg	Loggerhead	Fort Lauderdale	134604	125
H3.823.CM.F	823	Hatched Egg	Green Turtle	Fort Lauderdale	139100	117
H3.867.CM.F	867	Hatched Egg	Green Turtle	Fort Lauderdale	111563	114
H3.892.CC.F	892	Hatched Egg	Loggerhead	Fort Lauderdale	163214	159
H4.395.CC.H	395	Hatched Egg	Loggerhead	Hillsboro	84968	86
H5.395.CC.H	395	Hatched Egg	Loggerhead	Hillsboro	133163	145
H6.395.CC.H	395	Hatched Egg	Loggerhead	Hillsboro	135936	95
N.1166.CM.H	1166	Nest Sand	Green Turtle	Hillsboro	106640	427
N.1167.CM.H	1167	Nest Sand	Green Turtle	Hillsboro	149852	520
N.1168.CM.H	1168	Nest Sand	Green Turtle	Hillsboro	145635	261
N.127.CC.H	127	Nest Sand	Loggerhead	Hillsboro	116838	496
N.236.CC.H	236	Nest Sand	Loggerhead	Hillsboro	103352	814
N.242.CC.H	242	Nest Sand	Loggerhead	Hillsboro	128324	773
N.282.CC.H	282	Nest Sand	Loggerhead	Hillsboro	114036	751
N.322.CC.H	322	Nest Sand	Loggerhead	Hillsboro	127019	226
N.323.CC.H	323	Nest Sand	Loggerhead	Hillsboro	94186	434
N.392.CC.H	392	Nest Sand	Loggerhead	Hillsboro	174355	437
N.395.CC.H	395	Nest Sand	Loggerhead	Hillsboro	185642	462
N.456.CC.F	456	Nest Sand	Loggerhead	Fort Lauderdale	127266	422
N.457.CC.F	457	Nest Sand	Loggerhead	Fort Lauderdale	146810	341
N.463.CC.F	463	Nest Sand	Loggerhead	Fort Lauderdale	143510	435
N.463.CC.H	463	Nest Sand	Loggerhead	Hillsboro	123118	550
N.519.CC.F	519	Nest Sand	Loggerhead	Fort Lauderdale	134679	547
N.520.CC.F	520	Nest Sand	Loggerhead	Fort Lauderdale	71281	180
N.521.CC.F	521	Nest Sand	Loggerhead	Fort Lauderdale	170673	726
N.522.CC.F	522	Nest Sand	Loggerhead	Fort Lauderdale	73435	195
N.695.CM.H	695	Nest Sand	Green Turtle	Hillsboro	106954	466
N.696.CM.H	696	Nest Sand	Green Turtle	Hillsboro	130122	341
N.697.CC.F	697	Nest Sand	Loggerhead	Fort Lauderdale	107111	195
N.698.CC.F	698	Nest Sand	Loggerhead	Fort Lauderdale	159366	373
N.699.CC.F	699	Nest Sand	Loggerhead	Fort Lauderdale	108708	274
N.823.CM.F	823	Nest Sand	Green Turtle	Fort Lauderdale	169853	197
N.867.CM.F	867	Nest Sand	Green Turtle	Fort Lauderdale	152182	338
N.892.CC.F	892	Nest Sand	Loggerhead	Fort Lauderdale	139615	216
S.1166.CM.H	1166	Control Sand	Green Turtle	Hillsboro	103043	212
S.1167.CM.H	1167	Control Sand	Green Turtle	Hillsboro	153048	839
S.1168.CM.H	1168	Control Sand	Green Turtle	Hillsboro	115917	292
S.127.CC.H	127	Control Sand	Loggerhead	Hillsboro	75476	1158
S.236.CC.H	236	Control Sand	Loggerhead	Hillsboro	77044	1159
S.242.CC.H	242	Control Sand	Loggerhead	Hillsboro	84761	1322
S.282.CC.H	282	Control Sand	Loggerhead	Hillsboro	77507	1322
S.322.CC.H	322	Control Sand	Loggerhead	Hillsboro	79843	1122
S.323.CC.H	323	Control Sand	Loggerhead	Hillsboro	72290	1104
S.392.CC.H	392	Control Sand	Loggerhead	Hillsboro	77743	1253
S.395.CC.H	395	Control Sand	Loggerhead	Hillsboro	103204	1209
S.456.CC.F	456	Control Sand	Loggerhead	Fort Lauderdale	79914	1090
S.457.CC.F	457	Control Sand	Loggerhead	Fort Lauderdale	115285	1501
S.463.CC.F	463	Control Sand	Loggerhead	Fort Lauderdale	102250	1318
S.463.CC.H	463	Control Sand	Loggerhead	Hillsboro	112455	1644
S.519.CC.F	519	Control Sand	Loggerhead	Fort Lauderdale	61949	955
S.520.CC.F	520	Control Sand	Loggerhead	Fort Lauderdale	74880	115
S.521.CC.F	521	Control Sand	Loggerhead	Fort Lauderdale	81443	219
S.522.CC.F	522	Control Sand	Loggerhead	Fort Lauderdale	51353	916
S.695.CM.H	695	Control Sand	Green Turtle	Hillsboro	100271	227
S.696.CM.H	696	Control Sand	Green Turtle	Hillsboro	112269	476
S.697.CC.F	697	Control Sand	Loggerhead	Fort Lauderdale	117049	220
S.698.CC.F	698	Control Sand	Loggerhead	Fort Lauderdale	132441	267
S.699.CC.F	699	Control Sand	Loggerhead	Fort Lauderdale	82399	282
S.823.CM.F	823	Control Sand	Green Turtle	Fort Lauderdale	144798	192
S.867.CM.F	867	Control Sand	Green Turtle	Fort Lauderdale	139840	359
S.892.CC.F	892	Control Sand	Loggerhead	Fort Lauderdale	141714	351
U1.1166.CM.H	1166	Unhatched Egg	Green Turtle	Hillsboro	94444	121
U1.1167.CM.H	1167	Unhatched Egg	Green Turtle	Hillsboro	120812	144
U1.1168.CM.H	1168	Unhatched Egg	Green Turtle	Hillsboro	133301	122
U1.127.CC.H	127	Unhatched Egg	Loggerhead	Hillsboro	97504	47
U1.236.CC.H	236	Unhatched Egg	Loggerhead	Hillsboro	209641	115
U1.242.CC.H	242	Unhatched Egg	Loggerhead	Hillsboro	94836	45
U1.282.CC.H	282	Unhatched Egg	Loggerhead	Hillsboro	144418	89
U1.322.CC.H	322	Unhatched Egg	Loggerhead	Hillsboro	188918	75
U1.323.CC.H	323	Unhatched Egg	Loggerhead	Hillsboro	90928	101
U1.392.CC.H	392	Unhatched Egg	Loggerhead	Hillsboro	185816	108
U1.456.CC.F	456	Unhatched Egg	Loggerhead	Fort Lauderdale	201276	153
U1.457.CC.F	457	Unhatched Egg	Loggerhead	Fort Lauderdale	127385	151
U1.463.CC.F	463	Unhatched Egg	Loggerhead	Fort Lauderdale	98948	70
U1.463.CC.H	463	Unhatched Egg	Loggerhead	Hillsboro	149631	95
U1.519.CC.F	519	Unhatched Egg	Loggerhead	Fort Lauderdale	92312	28
U1.520.CC.F	520	Unhatched Egg	Loggerhead	Fort Lauderdale	80073	74
U1.521.CC.F	521	Unhatched Egg	Loggerhead	Fort Lauderdale	109555	98
U1.522.CC.F	522	Unhatched Egg	Loggerhead	Fort Lauderdale	88140	183
U1.695.CM.H	695	Unhatched Egg	Green Turtle	Hillsboro	138109	170
U1.696.CM.H	696	Unhatched Egg	Green Turtle	Hillsboro	93585	95
U1.697.CC.F	697	Unhatched Egg	Loggerhead	Fort Lauderdale	113833	86
U1.698.CC.F	698	Unhatched Egg	Loggerhead	Fort Lauderdale	128555	63
U1.699.CC.F	699	Unhatched Egg	Loggerhead	Fort Lauderdale	120485	92
U1.823.CM.F	823	Unhatched Egg	Green Turtle	Fort Lauderdale	128856	129
U1.867.CM.F	867	Unhatched Egg	Green Turtle	Fort Lauderdale	141268	93
U1.892.CC.F	892	Unhatched Egg	Loggerhead	Fort Lauderdale	109590	94
U2.1166.CM.H	1166	Unhatched Egg	Green Turtle	Hillsboro	129048	83
U2.1167.CM.H	1167	Unhatched Egg	Green Turtle	Hillsboro	131661	166
U2.1168.CM.H	1168	Unhatched Egg	Green Turtle	Hillsboro	151524	169
U2.127.CC.H	127	Unhatched Egg	Loggerhead	Hillsboro	82645	52
U2.236.CC.H	236	Unhatched Egg	Loggerhead	Hillsboro	120834	99
U2.242.CC.H	242	Unhatched Egg	Loggerhead	Hillsboro	73454	112
U2.282.CC.H	282	Unhatched Egg	Loggerhead	Hillsboro	96028	98
U2.322.CC.H	322	Unhatched Egg	Loggerhead	Hillsboro	107793	102
U2.323.CC.H	323	Unhatched Egg	Loggerhead	Hillsboro	79696	179
U2.392.CC.H	392	Unhatched Egg	Loggerhead	Hillsboro	228013	105
U2.456.CC.F	456	Unhatched Egg	Loggerhead	Fort Lauderdale	116602	102
U2.457.CC.F	457	Unhatched Egg	Loggerhead	Fort Lauderdale	164898	249
U2.463.CC.F	463	Unhatched Egg	Loggerhead	Fort Lauderdale	76160	206
U2.463.CC.H	463	Unhatched Egg	Loggerhead	Hillsboro	86925	87
U2.519.CC.F	519	Unhatched Egg	Loggerhead	Fort Lauderdale	99981	83
U2.520.CC.F	520	Unhatched Egg	Loggerhead	Fort Lauderdale	63424	97
U2.521.CC.F	521	Unhatched Egg	Loggerhead	Fort Lauderdale	197289	102
U2.522.CC.F	522	Unhatched Egg	Loggerhead	Fort Lauderdale	60187	101
U2.695.CM.H	695	Unhatched Egg	Green Turtle	Hillsboro	112345	82
U2.696.CM.H	696	Unhatched Egg	Green Turtle	Hillsboro	137847	107
U2.697.CC.F	697	Unhatched Egg	Loggerhead	Fort Lauderdale	99928	101
U2.698.CC.F	698	Unhatched Egg	Loggerhead	Fort Lauderdale	121995	98
U2.699.CC.F	699	Unhatched Egg	Loggerhead	Fort Lauderdale	90596	74
U2.823.CM.F	823	Unhatched Egg	Green Turtle	Fort Lauderdale	115456	120
U2.867.CM.F	867	Unhatched Egg	Green Turtle	Fort Lauderdale	148779	99
U2.892.CC.F	892	Unhatched Egg	Loggerhead	Fort Lauderdale	136076	65
U3.1166.CM.H	1166	Unhatched Egg	Green Turtle	Hillsboro	107034	48
U3.1167.CM.H	1167	Unhatched Egg	Green Turtle	Hillsboro	139796	91
U3.1168.CM.H	1168	Unhatched Egg	Green Turtle	Hillsboro	88387	124
U3.127.CC.H	127	Unhatched Egg	Loggerhead	Hillsboro	61215	69
U3.236.CC.H	236	Unhatched Egg	Loggerhead	Hillsboro	94793	198
U3.242.CC.H	242	Unhatched Egg	Loggerhead	Hillsboro	155937	65
U3.282.CC.H	282	Unhatched Egg	Loggerhead	Hillsboro	106699	109
U3.322.CC.H	322	Unhatched Egg	Loggerhead	Hillsboro	113420	94
U3.323.CC.H	323	Unhatched Egg	Loggerhead	Hillsboro	119792	46
U3.392.CC.H	392	Unhatched Egg	Loggerhead	Hillsboro	237925	112
U3.456.CC.F	456	Unhatched Egg	Loggerhead	Fort Lauderdale	210742	83
U3.457.CC.F	457	Unhatched Egg	Loggerhead	Fort Lauderdale	130341	136
U3.463.CC.F	463	Unhatched Egg	Loggerhead	Fort Lauderdale	126119	118
U3.463.CC.H	463	Unhatched Egg	Loggerhead	Hillsboro	129293	73
U3.519.CC.F	519	Unhatched Egg	Loggerhead	Fort Lauderdale	100580	100
U3.520.CC.F	520	Unhatched Egg	Loggerhead	Fort Lauderdale	89481	103
U3.521.CC.F	521	Unhatched Egg	Loggerhead	Fort Lauderdale	117780	51
U3.522.CC.F	522	Unhatched Egg	Loggerhead	Fort Lauderdale	103251	140
U3.695.CM.H	695	Unhatched Egg	Green Turtle	Hillsboro	104053	116
U3.696.CM.H	696	Unhatched Egg	Green Turtle	Hillsboro	128937	231
U3.697.CC.F	697	Unhatched Egg	Loggerhead	Fort Lauderdale	96902	114
U3.698.CC.F	698	Unhatched Egg	Loggerhead	Fort Lauderdale	120225	76
U3.699.CC.F	699	Unhatched Egg	Loggerhead	Fort Lauderdale	107036	73
U3.823.CM.F	823	Unhatched Egg	Green Turtle	Fort Lauderdale	148461	117
U3.867.CM.F	867	Unhatched Egg	Green Turtle	Fort Lauderdale	135124	192
U3.892.CC.F	892	Unhatched Egg	Loggerhead	Fort Lauderdale	143452	75

*Note*: The number of sequence reads per sample were quantified by QIIME‐2 (QIIME Sequence Reads) after sequencing on the Illumina MiSeq. Bacterial taxonomy counts (ASV Count) per sample were determined after the data contamination cleaning process.

**Table A2:** Alpha diversity pairwise significance results between sample types.

	Margalef's species richness			
	Loggerhead	Green Turtle	Fort Lauderdale	Hillsboro
Cloaca, control sand	<0.001	0.291	<0.001	<0.001
Cloaca, hatched egg	<0.001	0.997	0.089	<0.001
Cloaca, nest sand	0.030	0.044	0.358	0.089
Cloaca, unhatched egg	<0.001	1.000	0.007	<0.001
Control sand, hatched egg	<0.001	0.247	<0.001	<0.001
Control sand, nest sand	<0.001	0.924	<0.001	<0.001
Control sand, unhatched egg	<0.001	0.173	<0.001	<0.001
Hatched egg, nest sand	<0.001	0.020	<0.001	<0.001
Hatched egg, unhatched egg	0.136	0.999	0.747	0.166
Nest sand, unhatched egg	<0.001	0.011	<0.001	<0.001

	**Shannon diversity**			
	**Loggerhead**	**Green Turtle**	**Fort Lauderdale**	**Hillsboro**
Cloaca, control sand	<0.001	0.999	0.002	<0.001
Cloaca, hatched egg	0.004	0.952	0.393	0.004
Cloaca, nest sand	1.000	0.501	0.997	0.997
Cloaca, unhatched egg	<0.001	0.996	<0.001	<0.001
Control sand, hatched egg	<0.001	0.843	<0.001	<0.001
Control sand, nest sand	<0.001	0.347	0.006	<0.001
Control sand, unhatched egg	<0.001	1.000	<0.001	<0.001
Hatched egg, nest sand	0.004	0.732	0.185	0.019
Hatched egg, unhatched egg	<0.001	0.535	<0.001	<0.001
Nest sand, unhatched egg	<0.001	0.138	<0.001	<0.001

	**Inverse Simpson's diversity**			
	**Loggerhead**	**Green Turtle**	**Fort Lauderdale**	**Hillsboro**
Cloaca, control sand	–	–	1.000	0.973
Cloaca, hatched egg	–	–	0.998	1.000
Cloaca, nest sand	–	–	0.995	0.999
Cloaca, unhatched egg	–	–	0.237	0.001
Control sand, hatched egg	–	–	1.000	0.893
Control sand, nest sand	–	–	0.999	0.997
Control sand, unhatched egg	–	–	0.179	<0.001
Hatched egg, nest sand	–	–	1.000	0.989
Hatched egg, unhatched egg	–	–	0.008	<0.001
Nest sand, unhatched egg	–	–	0.081	<0.001

*Note*: Pairwise tests between the sample types were completed if the “best‐fit” GLMM retained the interactive variables (Sample.Type × Turtle.Species, Sample.Type × Beach). If an interaction was not found in the “best‐fit” model, no significant values were placed in the table (–). Pairwise tests using the "emmeans" package between the sample types were completed for each turtle species (loggerhead and green turtles) and at each beach (Fort Lauderdale and Hillsboro) for loggerhead samples only. Only significance values (*p* values) are shown in the table. Sample type comparisons that were not found to be significant are indicated in red.

**Table A3:** Alpha diversity pairwise significance results between turtle species and beaches for each sample type.

	Margalef's species richness						
	Cloaca		Control sand		Hatched egg	Nest sand	Unhatched egg
Loggerhead, Green Turtle	0.002		<0.001		0.560	0.056	0.633
Fort Lauderdale, Hillsboro	0.018		<0.001		0.326	0.001	0.8697

	**Shannon diversity**						
	**Cloaca**		**Control sand**		**Hatched egg**	**Nest sand**	**Unhatched egg**
Loggerhead, Green Turtle	0.013		<0.001		0.827	0.536	<0.001
Fort Lauderdale, Hillsboro	0.067		<0.001		0.763	0.2749	0.126

	**Inverse Simpson diversity**						
	**Cloaca**		**Control sand**		**Hatched egg**	**Nest sand**	**Unhatched egg**
Loggerhead, Green Turtle	–		–		–	–	–
Fort Lauderdale, Hillsboro	0.895		0.563		0.707	0.966	0.131

*Note*: Pairwise tests between turtle species (loggerhead and green turtles) and beaches (Fort Lauderdale and Hillsboro) for loggerhead samples only were completed for each sample type if the “best‐fit” GLMM retained the interactive variables (Sample.Type × Turtle.Species, Sample.Type × Beach). If an interaction was not found in the “best‐fit” model, no significant values were placed in the table (–). Only significance values (*p* values) are shown in the table. Sample type comparisons that were not found to be significant are indicated in red.

**Table A4:** One‐way ANOSIM pairwise comparisons for all sample types.

	Loggerhead		Green Turtle		Fort Lauderdale (Loggerheads only)		Hillsboro (Loggerheads only)	
Pairwise comparisons	R Statistic	*p* value	R Statistic	*p* value	R Statistic	*p* value	R Statistic	*p* value
Cloaca, hatched egg	0.784	0.001	0.687	0.001	0.618	0.001	0.989	0.001
Cloaca, nest sand	0.737	0.001	0.564	0.004	0.604	0.001	0.909	0.001
Cloaca, control sand	0.774	0.001	0.572	0.002	0.592	0.001	0.905	0.001
Cloaca, unhatched egg	0.719	0.001	0.266	0.034	0.49	0.001	0.98	0.001
Hatched egg, nest sand	0.329	0.001	0.318	0.026	0.287	0.008	0.292	0.002
Hatched egg, control sand	0.665	0.001	−0.026	0.519	0.377	0.001	0.997	0.001
Hatched egg, unhatched egg	0.345	0.001	0.132	0.001	0.262	0.001	0.421	0.001
Nest sand, control sand	0.496	0.001	0.275	0.003	0.235	0.002	0.985	0.001
Nest sand, unhatched egg	0.123	0.031	−0.079	0.693	0.072	0.196	0.132	0.096
Control sand, unhatched egg	0.609	0.001	−0.171	0.921	0.347	0.007	0.894	0.001

*Note*: ANOSIM R statistics comparing the mean of ranked dissimilarities between groups to the mean of ranked dissimilarities within groups were determined for all possible pairwise comparisons between sample types (cloaca, control sand, hatched egg, unhatched egg, and nest sand). ANOSIM analyses were completed separately by turtle species (loggerhead and green turtle) and beaches (Fort Lauderdale and Hillsboro) for loggerhead samples only. R statistics closer to “1.0” suggest dissimilarity between groups. Pairwise comparisons that were not found to be significant (*p* value) are indicated in red.

**Table A5:** SIMPER analyses for hatched and unhatched egg samples at the phylum‐level.

	Dissimilarity (%)	Relative abundance (%)		Top contributing bacterial phyla		*p* value
		Hatched egg | Unhatched egg		Phylum | Contribution (%)		
Loggerhead	31.52	52.97	65.05	Pseudomonadota	11.68	<0.001
		26.95	14.81	Bacteroidota	8.45	<0.001
		15.87	15.96	Bacillota	8.17	<0.001
		2.41	3.72	Actinomycetota	2.20	<0.001
Green Turtle	18.17	66.3	65.31	Pseudomonadota	7.01	<0.001
		11.78	19.75	Bacillota	6.27	0.002
		19.48	12.99	Bacteroidota	3.60	<0.001
Fort Lauderdale (Loggerhead only)	29.01	55.78	66.19	Pseudomonadota	11.10	0.001
		26.2	14.33	Bacteroidota	8.08	<0.001
		15.44	16.36	Bacillota	7.99	0.002
		1.77	2.47	Actinomycetota	1.21	0.011
Hillsboro (Loggerhead only)	34.20	49.87	63.48	Pseudomonadota	12.00	<0.001
		27.77	15.48	Bacteroidota	8.83	0.003
		16.34	15.41	Bacillota	8.34	0.003
		3.11	5.44	Actinomycetota	3.58	0.002

*Note*: SIMPER analyses between hatched and unhatched eggs were completed for each turtle species (loggerhead and green turtles) and at each beach (Fort Lauderdale and Hillsboro) for loggerhead samples only. ASVs were consolidated by phylum before analysis. The total percentage of dissimilarity between egg sample types, relative abundance per egg sample type, and top contributing bacterial phyla (with the percentage of contribution) were determined. Only the bacterial phyla with greater than 1% contribution were selected. The top bacterial phyla contributed a total of 30.50% to the total dissimilarity between hatched and unhatched eggs in loggerhead samples, 16.88% in green turtle samples, 28.38% in Fort Lauderdale samples, and 32.75% in Hillsboro samples.

**Table A6:** SIMPER analyses for hatched and unhatched egg samples at the genus‐level.

	Dissimilarity (%)	Relative abundance (%)		Top contributing bacterial genera		*p* value
		Hatched egg | Unhatched egg		Genus | Contribution (%)		
Loggerhead	77.00	1.31	24.80	*Pseudomonas*	11.85	0.001
		9.84	3.90	*Paenibacillus*	4.55	0.001
		10.95	4.58	*Sphingobacterium*	4.47	0.001
		4.46	7.95	*Alcaligenes*	4.28	0.012
		8.12	2.59	*Nitratireductor*	3.68	0.001
Green Turtle	43.68	0.72	11.23	*Pseudomonas*	5.31	0.001
		14.65	10.43	*Nitratireductor*	3.18	0.004
		14.32	9.73	*Sphingobacterium*	3.10	0.006
Fort Lauderdale (Loggerhead only)	72.39	1.40	25.49	*Pseudomonas*	12.18	0.001
		13.29	5.12	*Sphingobacterium*	4.91	0.001
		10.06	5.24	*Paenibacillus*	4.75	0.001
		9.66	3.51	*Nitratireductor*	4.08	0.002
		3.45	7.11	*Alcaligenes*	3.47	0.039
Hillsboro (Loggerhead only)	80.86	1.21	23.85	*Pseudomonas*	11.38	0.001
		5.57	9.11	*Alcaligenes*	5.17	0.116
		9.61	2.07	*Paenibacillus*	4.29	0.001
		8.39	3.83	*Sphingobacterium*	3.83	0.035
		6.01	2.56	*Flavobacterium*	3.21	0.234
		6.43	1.33	*Nitratireductor*	3.06	0.006

*Note*: SIMPER analyses between hatched and unhatched eggs were completed for each turtle species (loggerhead and green turtles) and at each beach (Fort Lauderdale and Hillsboro) for loggerhead samples only. The total percentage of dissimilarity between egg sample types, relative abundance per egg sample type, and top contributing bacterial genera (with the percentage of contribution) were determined. Only the bacterial taxa with greater than 3% contribution were selected. Contributing bacterial taxa that were not found to be significant are indicated in red. The top genera contributed a total of 28.83% to the total dissimilarity between hatched and unhatched eggs in loggerhead samples, 11.59% in green turtle samples, 29.39% in Fort Lauderdale samples, and 30.94% in Hillsboro samples.

**Table A7:** Relative abundances of bacteria identified in CAP analyses for hatched and unhatched eggs.

	Hatched egg		Unhatched egg	
Taxa	RA (%)	Variance (±%)	RA (%)	Variance (±%)
**Phylum**				
Bacteroidota	24.83	1.543	14.29	1.353
Verrucomicrobiota	0.27	0.003	0.03	<0.001
Myxococcota	0.53	0.005	0.09	<0.001
Bdellovibrionota	0.36	0.004	0.11	<0.001
Pseudomonadota	55.82	1.810	65.00	11.769
Bacillota	14.69	0.355	16.97	2.283
**Class**				
Alphaproteobacteria	32.47	1.019	16.04	0.671
Bacilli	13.72	0.331	9.69	1.645
Bacteroidia	24.82	1.543	14.29	1.353
Gammaproteobacteria	23.35	0.791	48.96	11.098
Clostridia	0.89	0.023	7.08	0.635
**Order**				
Sphingomonadales	3.09	0.055	0.77	0.004
Xanthomonadales	12.18	0.406	5.85	0.153
Sphingobacteriales	12.29	0.667	5.90	0.343
Rhizobiales	24.90	0.756	13.09	0.566
Pseudomonadales	1.25	0.011	21.01	7.658
**Family**				
Sphingomonadaceae	3.09	0.055	0.77	0.004
Xanthomonadaceae	12.07	0.405	5.82	0.153
Sphingobacteriaceae	12.28	0.667	5.90	0.343
Devosiaceae	1.77	0.014	0.76	0.007
Pseudomonadaceae	1.12	0.010	20.70	7.641
**Genus**				
*Sphingopyxis*	3.00	0.055	0.77	0.004
*Pseudonocardia*	0.51	0.002	0.15	<0.001
*Devosia*	0.85	0.006	0.21	0.001
*Cohnella*	0.24	<0.001	0.06	<0.001
*Pseudomonas*	1.10	0.010	19.29	7.337

*Note*: Canonical analysis of principal coordinates (CAP) using Bray‐Curtis dissimilarity matrices was used to determine which bacteria best categorize the hatched egg sample types from the unhatched egg sample types. CAP analyses were completed at various taxonomic rankings, from Phylum (top) to Genus (bottom). The relative abundance (RA [%]) of the bacteria identified and their variances (Variance [±%]) were compared between hatched (left two columns) and unhatched (right two columns) eggs. Instances where unhatched eggs had higher abundances are indicated by a red font. Within each taxonomic group, bacteria are organized by the strength of the CAP correlation in categorizing the egg sample types (greater strength at the top of the list).

**Table A8:** SIMPER analysis of sand microbiota between beaches.

Top contributing bacterial taxa	Hillsboro abundance	Fort Lauderdale abundance	Contribution (%)	*p* value
*Sphingobacterium* sp.	0.000	0.075	4.41	0.053
*Ochrobactrum* sp.	0.000	0.063	3.69	0.053
*Nitratireductor* sp.	0.000	0.055	3.22	0.041
*Nitratireductor* sp.	0.000	0.044	2.58	0.053
*Achromobacter* sp.	0.001	0.033	1.92	0.052
*Brevibacillus* sp.	0.000	0.027	1.61	0.053
*Stenotrophomonas* sp.	0.000	0.027	1.56	0.047
*Alcaligenes* sp.	0.005	0.023	1.26	0.043
*Stenotrophomonas* sp.	0.001	0.020	1.14	0.043

*Note*: SIMPER analyses between Fort Lauderdale and Hillsboro beach were completed for control sand sample types for loggerhead samples only. There was an 84.17% dissimilarity in microbiota between Fort Lauderdale and Hillsboro beach. Only the bacterial taxa with greater than 1% contribution were selected. Abundances of top contributing bacterial taxa at Hillsboro and Fort Lauderdale are listed before the contribution (%) of each bacterial taxon. Contributing bacterial taxa that were not found to be significant are indicated in red.

**Table A9:** Hatched and unhatched egg best‐fit generalized linear mixed‐effect model.

	Margalef's species richness	Shannon diversity	Inverse Simpson's diversity
Best‐fit model variables (dAIC)	Incubation.Length (4.7) High.Tide.Distance (4.0) Temperature.Bottom (8.2) Sample.Type × Turtle.Species (97.4) Sample.Type × Beach (64.1)	Clutch.Size (8.88) High.Tide.Distance (2.59) Temperature.Bottom (4.60) Sample.Type × Turtle.Species (76.37) Sample.Type × Beach (27.81)	Sorting.Coefficient (2.00) Sample.Type × Beach (4.98)
*R* ^2^ fixed effects alone	0.76	0.64	0.25
Model intercept	−61.24	0.37	0.96
Intercept [95% CI]; *t*‐value	[−154.86, 32.39]; *t* = −1.29	[−2.44, 3.17]; *t* = 0.26	[0.80, 1.12]; *t* = 11.95

*Note*: Generalized linear mixed‐effect models (GLMMs) were run to determine what metadata variables best explained variation in hatched and unhatched egg samples for each alpha diversity metric (Margalef's species richness, Shannon diversity, and Inverse Simpson's diversity). The best‐fit model variables were determined using term selection by AIC score and incorporated a random effect (Nest Number) to account for pseudo‐replication. The score difference between the full model and the model without the listed variable (dAIC) is also listed. An *R* ^2^ value for the fixed effects alone is listed to show the explanatory power of the model. The intercept of the model along with its 95% confidence intervals (CI) and *t*‐value are also listed. All GLMMs had different variables affecting their variance with varying explanatory power.

**Table A10:** Hatched and unhatched egg BIO‐ENV correlations for each turtle species and beach.

	Loggerhead	Green Turtle	Fort Lauderdale (Loggerhead only)	Hillsboro (Loggerhead only)
**BIO‐ENV correlation**	0.228	0.211	0.363	0.269
**Environmental variables**	Clutch size Hatch success Chamber depth High tide distance Conductivity (Bottom) Sand grain size	Incubation length Clutch size Temperature (Bottom) pH (Bottom)	High tide distance Sand grain size	Longitude Hatch success High tide distance Dune distance Temperature (Side) Conductivity (Side)
**Individual environmental variable (correlation)**	Sand grain size (0.152)	Incubation length (0.167)	Sand grain size (0.286)	Conductivity (Side) (0.209)
***p* value**	0.001	0.018	0.001	0.001

*Note*: BIO‐ENV correlations were determined for hatched and unhatched eggs for each turtle species (loggerhead and green turtles) and each beach (Fort Lauderdale and Hillsboro) for loggerhead samples only. The highest correlation (BIO‐ENV correlation) and the set of environmental variables that go with it are given in the first two rows. The individual environmental variable with the highest correlation is also listed with its correlation in parentheses. All BIO‐ENV analyses found significant correlations (*p* < 0.05) between the identified environmental variables and patterns in bacterial community composition.
